# Certainty of the Global Burden of Disease 2019 Modelled Prevalence Estimates for Musculoskeletal Conditions: A Meta-Epidemiological Study

**DOI:** 10.3389/ijph.2023.1605763

**Published:** 2023-05-31

**Authors:** Javier Muñoz Laguna, Milo A. Puhan, Fernando Rodríguez Artalejo, Robby De Pauw, Grant M. A. Wyper, Brecht Devleesschauwer, João V. Santos, Cesar A. Hincapié

**Affiliations:** ^1^ Department of Preventive Medicine, Public Health and Microbiology, School of Medicine, Universidad Autónoma de Madrid, Madrid, Spain; ^2^ Epidemiology, Biostatistics and Prevention Institute, University of Zurich, Zurich, Switzerland; ^3^ University Spine Centre Zurich (UWZH), Balgrist University Hospital, University of Zurich, Zurich, Switzerland; ^4^ EBPI-UWZH Musculoskeletal Epidemiology Research Group, University of Zurich and Balgrist University Hospital, Zurich, Switzerland; ^5^ CIBERESP (CIBER of Epidemiology and Public Health), Madrid, Spain; ^6^ IMDEA Food Institute, CEI UAM + CSIC, Madrid, Spain; ^7^ Department of Epidemiology and Public Health, Sciensano, Brussels, Belgium; ^8^ Department of Rehabilitation Sciences, Ghent University, Ghent, Belgium; ^9^ School of Health & Wellbeing, University of Glasgow, Glasgow, United Kingdom; ^10^ Place and Wellbeing Directorate, Public Health Scotland, Glasgow, United Kingdom; ^11^ Department of Translational Physiology, Infectiology and Public Health, Ghent University, Merelbeke, Belgium; ^12^ MEDCIDS—Department of Community Medicine, Information and Health Decision Sciences, Faculty of Medicine, University of Porto, Porto, Portugal; ^13^ CINTESIS—Centre for Health Technology and Services Research, Porto, Portugal; ^14^ Public Health Unit, ACES Grande Porto V – Porto Ocidental, ARS Norte, Porto, Portugal

**Keywords:** global burden of disease, epidemiology, prevalence, musculoskeletal disorders, low back pain, neck pain, knee osteoarthritis, GRADE approach

## Abstract

**Objectives:** To describe and assess the risk of bias of the primary input studies that underpinned the Global Burden of Disease Study (GBD) 2019 modelled prevalence estimates of low back pain (LBP), neck pain (NP), and knee osteoarthritis (OA), from Australia, Brazil, Canada, Spain, and Switzerland. To evaluate the certainty of the GBD modelled prevalence evidence.

**Methods:** Primary studies were identified using the GBD Data Input Sources Tool and their risk of bias was assessed using a validated tool. We rated the certainty of modelled prevalence estimates based on the GRADE Guidelines 30―the GRADE approach for modelled evidence.

**Results:** Seventy-two primary studies (LBP: 67, NP: 2, knee OA: 3) underpinned the GBD estimates. Most studies had limited representativeness of their study populations, used suboptimal case definitions and applied assessment instruments with unknown psychometric properties. The certainty of modelled prevalence estimates was low, mainly due to risk of bias and indirectness.

**Conclusion:** Beyond the risk of bias of primary input studies for LBP, NP, and knee OA in GBD 2019, the certainty of country-specific modelled prevalence estimates still have room for improvement.

## Introduction

Since its first publication in 1993, the Global Burden of Disease Study (GBD) has become a reference in the estimation of disease burden, including for musculoskeletal (MSK) disorders [1]. According to GBD 2019, MSK disorders ranked globally first in terms of years lived with disability (YLD) and sixth in disability adjusted life years (DALY) across all diseases [1]. While prevalence estimates vary [2–4], it has been suggested that one in three people live with a chronic, painful MSK condition [5]. Among MSK disorders, low back pain (LBP), neck pain (NP), and knee osteoarthritis (OA) are especially burdensome, motivating focused GBD analyses and calls for action [6, 7].

Due to the ambitious aim of estimating prevalence, and other disease metrics impacted by prevalence (i.e., YLD, DALY) for 204 countries and territories between 1990 and 2019, GBD 2019 estimates rely on modelling data, especially when primary data are scarce [8, 9]. This dependence on complex modelling techniques is accentuated in low- and middle-income countries, where the lack of infrastructure limits periodic epidemiologic studies and where MSK disorders are often overlooked [10]. Given the proportion of people not seeking care for MSK pain, burden estimation faces specific challenges due to the lack of suitability of routine administrative data sources (i.e., hospital and claims data) for this purpose, and its unique DALY profile, which is almost exclusively dependent on YLD. Despite a recent study on the completeness of primary LBP prevalence data in GBD 2017 [11], little is known about the characteristics and risk of bias of the primary data input studies that underpinned the GBD 2019 modelled prevalence estimates of LBP, NP, and knee OA, as well as the certainty of these estimates.

As GBD metrics might guide health decision-making and resource allocation, the application of approaches to rate the certainty of GBD modelled estimates for MSK conditions is relevant. Complementary to advances in quality rating systems in GBD (i.e., star rating in Burden of Proof studies) [12], the Grading of Recommendations, Assessment, Development and Evaluation (GRADE) working group offers an intuitive approach to rate the certainty of a body of evidence and, thus, the confidence we can place on it, according to risk of bias, inconsistency, indirectness, imprecision, reporting bias, magnitude of an effect, dose-response relation, and the direction of residual confounding [13]. Beyond the wide use of GRADE in systematic reviews and clinical practice guidelines, important variations have emerged for other types of evidence [14], including modelling studies [15]. Following recommendations to use GRADE for modelling studies to improve World Health Organization (WHO) guidelines [16], this framework has yet to be applied to GBD epidemiologic estimates.

### Objectives

This meta-epidemiological work aims to: 1) describe and assess the risk of bias of the primary input studies that underpinned the GBD 2019 modelled prevalence estimates of LBP, NP, and knee OA, from five countries (Australia, Brazil, Canada, Spain, and Switzerland), and 2) evaluate the certainty of the modelled prevalence evidence using GRADE.

## Methods

### Data Identification and Retrieval

We adhered to applicable items of the guidelines for reporting meta-epidemiological research (Supplementary Datasheet S1) [17]. For our search strategy―carried out between December 2021 and April 2022―one reviewer independently used the GBD 2019 Data Input Sources Tool to systematically identify all primary data input studies. These were operationalised as peer-reviewed epidemiological studies and non-peer-reviewed national and international survey reports that informed the GBD 2019 modelled prevalence estimates of LBP, NP, and knee OA for Australia, Brazil, Canada, Spain, and Switzerland between 1990 and 2019 (Supplementary Datasheet S1 p 3). Primary data input studies were eligible if they were indexed in the 2019 Data Input Sources Tool and the Supplementary Appendix 1 of the GBD 2019 publication [1]. The GBD 2019 Supplementary Appendix 1 provides a rationale for the specific study characteristics used as eligibility criteria in the prevalence estimation of LBP, NP, and knee OA. We did not consider additional databases or internet searches, and we did not contact experts beyond those in the author group to identify additional studies. We selected the three most prevalent MSK pain conditions investigated in the GBD 2019 Study (LBP, NP, and knee OA) and five countries covering four world continents. Although these are primarily high-income countries, our country selection took into consideration the trade-off between the scope of the work, external validity, and our limited resource constraints. In addition, our pragmatic decision to select Australia, Brazil, Canada, Spain, and Switzerland considered their diverse healthcare systems, as well as the language skills and relevance of these countries to the authors. Before strategic selection and protocol specification, we conducted a preliminary search and gained information on the availability of at least some primary data coverage for the three most prevalent MSK pain conditions the GBD 2019 Study for these five territories. We excluded other sources of input data, mainly opportunistic surveys, and insurance claims, as they were not identifiable through the GBD website or GBD 2019 Study Supplementary Appendices. Our protocol was registered on the Open Science Framework [18].

All primary input studies were independently retrieved for risk of bias assessment and data extraction. When primary input studies were not accessible by conventional means (i.e., via electronic academic journals or scientific search engines), alternative approaches were tried, including searching open-source repositories and directly contacting primary study authors by email. In the case of the World Health Survey and the Swiss Household Panel Survey, two formal applications were completed and granted via the WHO Multi-Country Studies Data Archive (an open-access WHO repository) and the SWISSUbase system (a cross-disciplinary Swiss repository), respectively (Supplementary Datasheet S1 p 3).

### Descriptive Analysis

One reviewer independently tabulated key information from primary input studies, including their sampling frame (national vs. subnational), sampling design, sample size, response proportion, years covered, sample age range, proportion of female participants, case definition for LBP, NP, or knee OA with applicable prevalence periods, and prevalence estimates with 95% confidence intervals (CIs). Prevalence periods were extracted from the ‘Methods’ section or from instruments referenced in primary input studies. For instance, if a primary input study asked about pain lasting 1 day or longer during the preceding month in the lumbar area, this was considered a one-month prevalence period. Prevalence was operationalised as the proportion of cases of LBP, NP, or knee OA in the included study populations. All extractable variables were prespecified in our protocol. When only strata-specific prevalence estimates were reported in primary studies (i.e., overall prevalence estimates not reported), we combined these into a single age and sex combined point estimate, conditional on having enough data granularity for the given study (total N, N for each individual age and sex stratum, and prevalence estimate for each stratum). Similarly, when interval estimates were not reported for prevalence point estimates, we derived 95% CIs, if possible, using Wald standard errors accounting for sampling design effects (see Supplementary Datasheet S1 p 4 for details) [2]. We did not use additional processes for manipulating data.

### Risk of Bias Assessment

To assess risk of bias in primary input studies, we used a tool validated by Hoy et al. (Supplementary Datasheet S1 pp 5–8) [19], which has shown high interrater reliability in population-based cross-sectional studies of LBP [2], NP [20], and knee OA [21]. Ten criteria were rated as “low” or “high” risk of bias for each study: 1) representativeness of the target population in relation to the national population, 2) representativeness of the sampling frame in relation to the target population, 3) inclusion of random selection or a census, 4) likelihood of non-response bias, 5) collection of data directly from the subjects (as opposed to a proxy), 6) use of an acceptable case definition with anatomical specification, 7) use of a study instrument to measure prevalence with known reliability and validity, 8) uniformity in the mode of data collection, 9) appropriateness of the length of the shortest prevalence period for the parameter of interest, and (10) appropriateness of the numerator and denominator for the prevalence estimate. An additional summary item, added to the risk of bias visualizations [22], indicated how likely it would be for further research to change the confidence in the value of an observed prevalence estimate derived from a primary study. To ensure consistent and reliable risk of bias ratings and judgments, we conducted a training and calibration phase for the use of the tool and assessed interrater reliability. Two reviewers (JML and CAH) independently evaluated 20 (28%) of the 72 studies (randomly selected) and obtained 92% agreement on ratings and a *kappa* of 0.82 (95% CI, 0.73–0.91), suggesting high agreement beyond chance.

### Certainty of GBD Prevalence Estimates

We performed a certainty assessment of the GBD 2019 modelled prevalence estimates using GRADE Guidelines 30—the GRADE approach for modelled evidence [15]. GRADE Guidelines 30 maintains general concepts of the GRADE approach and proposes a framework for selecting the best available evidence from one or more models to inform healthcare decisions. We selected these guidelines due to their applicability to health decision-making. Even though no specific guidance is available for applying GRADE to modelled prevalence estimates, GRADE Guidelines 30 allowed the identification of an existing model (GBD 2019) that *a priori* provided the highest certainty evidence for modelled prevalence of LBP, NP, and knee OA between 1990 and 2019. Due to the nature of our modelled outcome of interest, we performed assessments based on the GRADE criteria of risk of bias, inconsistency, indirectness, and imprecision of modelled prevalence estimates [15].

GRADE application began with the assessment of risk of bias, by which country-specific modelled prevalence estimates were evaluated based on the credibility of the disease-specific model (i.e., conceptualization, structure, calibration, and other factors) and the certainty of evidence in each of its inputs (i.e., whether model inputs represented the entire body of relevant evidence satisfying clear prespecified criteria). We then qualitatively assessed modelled prevalence estimates for indirectness based on two subdomains: indirectness of model inputs with respect to the model (based on the presence of country-specific primary input studies), and indirectness of model outputs with respect to the national MSK modelled estimation of interest. The third step involved assessing inconsistency of modelled estimates, by examining the variability in the modelled prevalence estimates over the study period. Lastly, we evaluated modelled estimates for imprecision by judging the width of modelled uncertainty intervals. Due to the nature of our modelled outcome of interest, we did not consider additional GRADE criteria (i.e., dose-response gradient, large magnitude of effect, and effect of an opposite direction of plausible residual confounding) to be applicable.

### Patient and Public Involvement

Patients and members of the public were not involved in this study due to resource limitations. Nonetheless, we intend to involve the public in disseminating our results, including via social media platforms, newsletters, and conferences.

## Results

### Data Identification, Availability, and Coverage Between 1990 and 2019

**FIGURE 1:**
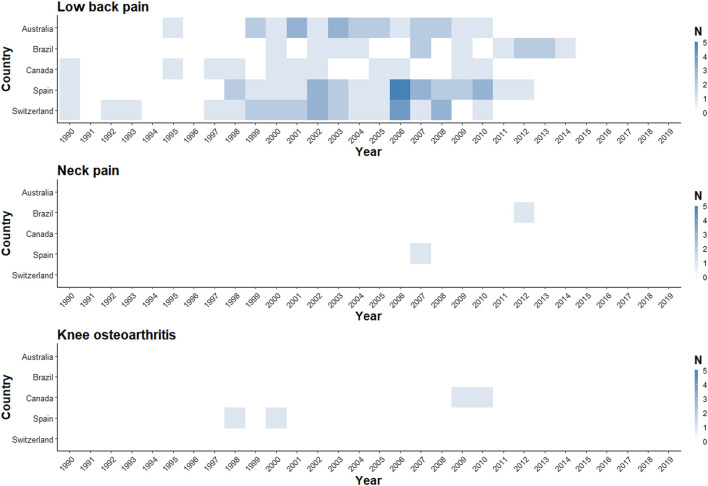
Number of low back pain, neck pain, and knee osteoarthritis primary input studies informing year-specific modelled prevalence estimates (Global Burden of Disease Study 2019—Australia, Brazil, Canada, Spain, and Switzerland [1990 to 2019]).

We identified 72 primary data input studies (LBP: 67, NP: 2, knee OA: 3; Australia: 12; Brazil: 11; Canada: 8; Spain: 22; Switzerland: 19) in the GBD 2019 Data Input Sources Tool. After accounting for two studies providing input on LBP in two countries [23, 24], and two studies informing two MSK conditions in the same country [25, 26], 68 distinct primary input studies were identified and assessed for eligibility (Table 1; Supplementary Datasheet S1 p 9). Full texts, primary reports, or microdata (to obtain prevalence estimates when full texts or primary reports were not available) were retrieved for all studies. The World Health Survey for Australia was not available in the WHO central data catalogue—microdata and a report were provided by the WHO Department of Noncommunicable Diseases [33]. For the Swiss Household Panel Survey, since no periodic national reports were published, information was retrieved from microdata and other methodological publications [89, 90]. One of the input studies for Australia was a misclassified Austrian study [42]. Overall, Spain and Switzerland had the highest number of primary input studies for LBP (N = 19 each), while Canada had the lowest number (N = 7). Only the years 2000 and 2002 had at least one primary input study in all five included countries for LBP, and most of the studies were clustered between 1999 and 2010. Eight of the 30 included years did not have primary data coverage in any of the countries for LBP. For NP, only Spain and Brazil had primary data input studies, while for knee OA, this was the case for Spain and Canada (Figure 1; Supplementary Datasheet S1 pp 10–12).

**TABLE 1:** Summary characteristics of primary data input studies informing Global Burden of Disease Study 2019 modelled epidemiologic estimates of low back pain, neck pain, and knee osteoarthritis in Australia, Brazil, Canada, Spain, and Switzerland, 1990–2019.^a^

Study	RoB	N	Period	Prevalence %	Study	RoB	N	Period	Prevalence %
LBP — Australia					LBP — Spain (cont.)				
NHS 1995 [27, 28]	Mod	53,828	Point	17.6 (17.0–18.2)	Miró 2007 [75]	Low	592	3 mo	43.9 (39.9–47.9)
NHS 2001 [29, 30]	Mod	26,863	Point	21.0 (20.4–21.6)	Pellisé 2009 [23]	Low	1,470	1 mo	39.8 (36.3–43.3)
SDAC 2003 [31, 32]	Mod	41,386	12 mo	3.1 (2.9–3.3)	HBSC 2010 [76]	Mod	11,230	6 mo	38.1 (36.8–39.4)
WHS 2003 [33]	Mod	1,846	1 mo	44.1 (40.8–47.3)	Fernández de las Peñas 2011 [77, 78]	Low	29,478	12 mo	19.9 (19.3–20.5)
Walker 2004 [34]	Low	1,913	Point	25.6 (23.7–27.6)	Balagué 2012 [24]	Low	1,470	1 mo	39.8 (36.3–43.3)
NHS 2004–2005 [35, 36]	Mod	25,906	Point	16.4 (15.8–17.0)	Eurobarometer 2012 [79, 80]	Low	1,026	1 wk	7.9 (6.0–9.8)
Grimmer 2006 [37]	Low	434	1 wk	7.1 (3.7–10.5)	Jiménez Sánchez 2012 [26, 81]	Mod	12,190	12 mo	11.1 (10.5–11.7)
NHS 2007–2008 [38, 39]	Mod	20,788	Point	14.5 (13.7–15.3)	Rodríguez Oviedo 2012 [82]	High	1,403	12 mo	25.9 (22.7–29.1)
SDAC 2009 [40, 41]	Mod	73,683	12 mo	2.9 (2.8–3.0)	Vargas Prada 2013 [83]	Mod	1,105	12 mo	63.6 (59.6–67.6)
HBSC Austria 2010 [42]	Mod	6,493	6 mo	37.1 (35.4–38.8)	Mesas 2014 [84, 85]	Low	8,283	12 mo	14.1 (13.4–14.8)
Broom 2012 [43]	Mod	9,820	12 mo	54.8 (53.8–55.8)	Koyanagi 2015 [86, 87]	Mod	3,625	1 mo	45.1 (42.2–48.0)
O'Sullivan 2012 [44]	Mod	1,288	1 mo	12.3 (9.8–14.8)	LBP — Switzerland				
LBP — Brazil					Balagué 1994 [88]	Mod	1,716	1 wk	12.0 (10.4–13.6)
WHS 2003 [45]	Low	4,999	1 mo	52.6 (50.6–54.6)	HBSC 1998 [56]	Mod	5,520	6 mo	8.0–23.0 (95% CIs NR)
Silva 2004 [46]	Mod	3,182	Point	4.2 (3.5–4.9)	SHP 1999–2000 [89, 90]	Mod	7,799	12 mo	34.2 (33.1–35.3)
Mendoza-Sassi 2006 [47]	Mod	1,259	2 mo	35.1 (32.5–37.7)	Santos-Eggimann 2000 [91]	Mod	3,227	12 mo	9.5–38.5 (5.4–48.0)^b^
Blay 2007 [48]	Mod	6,961	6 mo	43.2 (41.9–44.3)	SHP 2000–2001 [89, 90]	Mod	7,073	12 mo	38.7 (37.6–39.8)
De Vitta 2011 [49]	Low	1,236	12 mo	19.5 (17.3–21.7)	SHP 2001–2002 [89, 90]	Mod	6,601	12 mo	38.4 (37.2–39.6)
Ferreira 2011 [50]	Low	972	12 mo	40.0 (36.9–43.2)	HBSC 2002 [59, 92]	Mod	9,275	6 mo	38.6 (37.2–40.0)
Onofrio 2012 [51]	Low	1,233	1 mo	13.7 (11.8–15.6)	SHP 2002–2003 [89, 90]	Mod	5,700	12 mo	37.8 (36.5–39.1)
Meziat Filho 2015 [52]	High	1,102	1 mo	28.6 (25.9–31.2)	SHP 2003–2004 [89, 90]	Mod	5,220	12 mo	35.8 (34.5–37.1)
Depintor 2016 [53]	Low	826	1 mo	18.4 (15.8–21.2)	SHP 2004–2005 [89, 90]	Mod	8,065	4 wk	44.8 (43.7–45.9)
Noll 2016 [54]	Mod	1,597	3 mo	55.7 (53.1–58.3)	HBSC 2006 [92, 62, 93]	Mod	9,507	6 mo	42.4 (41.0–43.8)
LBP — Canada					SHP 2006–2007 [89, 90]	Mod	6,657	4 wk	44.7 (43.5–45.9)
Liira 1996 [55]	Mod	38,540	Point	7.8 (7.2–8.4)	SHP 2007–2008 [89, 90]	Mod	6,979	4 wk	45.3 (44.1–46.5)
HBSC 1997–1998 [56, 57]	Mod	6,215	6 mo	49.7 (47.9–51.5)	SHP 2008–2009 [89, 90]	Mod	6,903	4 wk	44.8 (43.6–46.0)
Cassidy 1998 [58]	Low	1,131	Point	28.7 (26.1–31.4)	Pellisé 2009 [23]	Low	1,470	1 mo	39.8 (36.3–43.3)
HBSC 2002 [59, 60]	Mod	4,458	6 mo	41.2 (39.2–43.2)	HBSC 2010 [92, 64, 94]	Mod	9,886	6 mo	41.8 (40.4–43.2)
Currie 2004 [61]	Mod	118,533	12 mo	8.9 (8.7–9.1)	Erne 2011 [95]	Mod	189	1 mo	13.8 (6.8–20.8)
HBSC 2005 [62, 63]	Mod	9,670	6 mo	46.6 (45.2–48.0)	Kolb 2011 [96]	Mod	3,881	12 mo	33.2 (31.7–34.7)
HBSC 2010 [64, 65]	Mod	26,078	6 mo	44.0 (43.1–44.9)	Balagué 2012 [24]	Low	1,470	1 mo	39.8 (36.3–43.3)
LBP — Spain					NP — Brazil				
Ballina García 1994 [66]	Mod	702	12 mo	28.2 (23.9–31.0)	Genebra 2017 [97]	Low	600	12 mo	20.3 (17.3–23.7)
Carmona 2001 [25, 67]	Low	2,192	Point	14.8 (12.2–17.4)	NP — Spain				
Català 2002 [68]	Mod	5,000	Point	11.9 (10.6–13.2)	Jiménez Sánchez 2012 [26, 81]	Mod	12,190	12 mo	6.0 (5.6–6.4)
HBSC 2002 [59, 69]	Mod	13,552	6 mo	42.5 (41.3–43.7)	Knee OA — Canada				
WHS 2002–2003 [70]	Mod	6,275	1 mo	35.1 (33.4–36.8)	Plotnikoff 2015 [98, 99]	Mod	4,733	Point	10.5 (9.3–11.7)
HBSC 2006 [62, 71]	Mod	21,811	6 mo	38.6 (37.7–39.5)	Knee OA — Spain				
Pinto Meza 2006 [72]	Mod	2,121	12 mo	14.7 (12.3–17.1)	Carmona 2001 [25, 67]	Low	2,192	1 mo	10.2 (8.5–11.9)
Demyttenaere 2007 [73, 74]	Low	2,121	12 mo	14.7 (12.6–16.8)	Fernández López 2008 [100]	Low	2,192	1 mo	10.2 (7.9–12.5)

^a^ There were no primary data input studies for neck pain from Australia, Canada, and Switzerland, as well as no primary data input studies for knee OA from Australia, Brazil, and Switzerland. Prevalence estimates are reported as point estimates with 95% CIs.

^b^ Range of strata-specific prevalence estimates and confidence intervals for different age groups and genders; overall total prevalence estimate not reported.

CI, confidence interval; HBSC, Health Behaviour in School-aged Children; LBP, low back pain; mo, months; Mod, moderate; N, study size; NHS, National Health Survey; NP, neck pain; NR, not reported; OA, osteoarthritis; Period, prevalence period; RoB, overall study risk of bias; SDAC, Survey of Disability, Ageing and Carers; SHP, Swiss Household Panel; WHS, World Health Survey; wk, weeks.

### Characteristics of Primary Input Studies

Table 1 summarises key characteristics of the primary input studies. The included studies had between 189 and 118,533 participants, with most studies (61 of 67) having more than 1,000 participants. There were variations in the sampling frame of studies for LBP (see detailed evidence table in Supplementary Datasheet S1 pp 13–32), with more than a third of them (25 of 67) having a subnational focus. Most studies for LBP used stratified, clustered, or a multistage combination of probabilistic sampling approaches, although 9 of 67 did not specify the strategy. The proportion of respondents was adequate for most studies, although 2 of 67 did not reach a 50% response proportion. The variability of LBP case definitions was striking, with different recall periods, chronicity, anatomical pain locations, and activity-limiting considerations. Primary data prevalence estimates varied considerably between studies and countries.

The two primary data input studies for NP included subnational sampling frames and used probabilistic multistage sampling (detailed evidence table in Supplementary Datasheet S1 p 33) [26, 97]. However, these two studies differed substantially in their sample size (12,190 participants vs. 600) and proportion of respondents (38% vs. 94%). They also differed in their case definitions, since one study did not indicate the anatomical location of the neck and asked participants for medical confirmation [26], while the other specified the anatomical location between the occiput and the third thoracic vertebra and used a validated tool [97]. Their NP one-year period prevalence estimates also varied substantially (Table 1; Supplementary Datasheet S1 p 33).

Two of the three primary studies for knee OA included national samples and used stratified multistage cluster sampling [25, 100], while the remaining study included a national sample but used a random digit dial strategy (detailed evidence table in Supplementary Datasheet S1 p 34) [98]. Differences were observed in response proportions, prevalence periods, and case definitions. The prevalence of knee OA was similar in the three studies (Table 1; Supplementary Datasheet S1 p 34).

### Risk of Bias of Primary Input Studies

The main criteria items in Hoy’s tool to introduce bias in primary studies (items 1, 6, and 7) corresponded to target populations not representing national populations in terms of key demographic characteristics (limited generalizability: 30 of 72 studies), suboptimal case definitions (no anatomical location specification: 49 of 72 studies), and the use of study instruments without demonstrated reliability or validity (prone to misclassification: 47 of 72 studies). Based on the overall summary rating, 48 of 72 studies were rated as moderate risk of bias, suggesting that further research may have an important impact on the confidence placed in their observed prevalence estimates (Supplementary Datasheet S1 pp 35–42).

### Certainty of GBD Modelled Prevalence Estimates—GRADE Assessment

**FIGURE 2:**
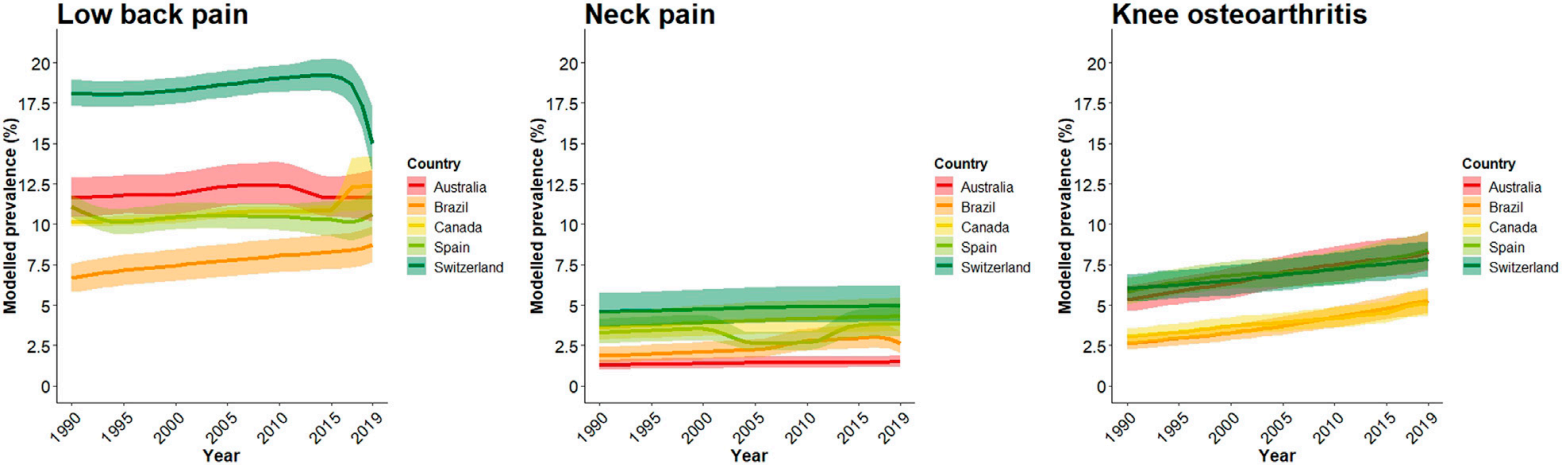
Modelled prevalence trends with 95% uncertainty intervals for low back pain, neck pain, and knee osteoarthritis (Global Burden of Disease Study 2019–Australia, Brazil, Canada, Spain, and Switzerland [1990 to 2019]).

With all modelled prevalence estimates (Figure 2; Supplementary Datasheet S1 pp 43–48) starting by default at “high certainty,” we downgraded them two levels for risk of bias, which made them transition to “low certainty” (Table 2). This downgrade was justified due to concerns about the structure of the models and the lack of certainty of evidence in each of their model inputs. In countries without identifiable primary input studies, we downgraded modelled prevalence estimates one certainty level due to indirectness of model inputs with respect to the model. Having found significant unexplained variability in modelled LBP prevalence outputs in Switzerland between 2016 and 2019 and NP prevalence outputs in Spain between 2001 and 2014, we downgraded one additional certainty level due to inconsistency in these two cases. We did not downgrade modelled estimates for imprecision since uncertainty intervals around point estimates were judged to be precise, regardless of the amount of primary input studies. Taken together, the certainty of modelled LBP, NP, and knee OA prevalence estimates in Australia, Brazil, Canada, Spain, and Switzerland ranged between very low and low, with greater certainty found for LBP (Table 2; see detailed GRADE rating explanations in Supplementary Datasheet S1 pp 49–54).

**TABLE 2:** Summary certainty assessment for modelled prevalence estimates of low back pain, neck pain, and knee osteoarthritis following Grading of Recommendations, Assessment, Development and Evaluation guidelines 30 (Global Burden of Disease Study 2019–Australia, Brazil, Canada, Spain, and Switzerland [1990 to 2019]).

Country Condition	GRADE criteria				Range of modelled prevalence (95% UI)	Overall certainty
	Risk of bias	Inconsistency	Indirectness	Imprecision		
Australia						
LBP	Very serious ↓↓	Not serious	Not serious	Not serious	11.6 to 12.4 (10.2–13.9)	Low^a^ ^,^ ^b^ ⊕⊕○○
NP	Very serious ↓↓	Not serious	Serious ↓	Not serious	1.3 to 1.5 (1.0–1.9)	Very low^a^ ^,^ ^b^ ^,^ ^c^⊕○○○
Knee OA	Very serious ↓↓	Not serious	Serious ↓	Not serious	5.4 to 8.3 (4.6–9.5)	Very low^a^ ^,^ ^b^ ^,^ ^c^⊕○○○
Brazil						
LBP	Very serious ↓↓	Not serious	Not serious	Not serious	6.7 to 8.7 (5.8–9.9)	Low^a^ ^,^ ^b^ ⊕⊕○○
NP	Very serious ↓↓	Not serious	Not serious	Not serious	1.9 to 3.0 (1.5–3.9)	Low^a^ ^,^ ^b^ ^,^ ^c^ ⊕⊕○○
Knee OA	Very serious ↓↓	Not serious	Serious ↓	Not serious	2.7 to 5.4 (2.3–6.1)	Very low^a^ ^,^ ^b^ ^,^ ^c^⊕○○○
Canada						
LBP	Very serious ↓↓	Not serious	Not serious	Not serious	10.1 to 12.4 (9.8–14.2)	Low^a^ ^,^ ^b^ ⊕⊕○○
NP	Very serious ↓↓	Not serious	Serious ↓	Not serious	3.6 to 4.3 (2.9–5.4)	Very low^a^ ^,^ ^b^ ^,^ ^c^⊕○○○
Knee OA	Very serious ↓↓	Not serious	Not serious	Not serious	3.0 to 5.1 (2.6–5.9)	Low^a^ ^,^ ^b^ ⊕⊕○○
Spain						
LBP	Very serious ↓↓	Not serious	Not serious	Not serious	10.1 to 11.1 (9.0–12.2)	Low^a^ ^,^ ^b^ ⊕⊕○○
NP	Very serious ↓↓	Serious ↓	Not serious	Not serious	2.6 to 3.9 (2.2–4.8)	Very low^a^ ^,^ ^b^ ^,^ ^d^⊕○○○
Knee OA	Very serious ↓↓	Not serious	Not serious	Not serious	5.8 to 8.4 (5.0–9.6)	Low^a^ ^,^ ^b^ ⊕⊕○○
Switzerland						
LBP	Very serious ↓↓	Serious ↓	Not serious	Not serious	15.0 to 19.2 (13.1–20.3)	Very low^a^ ^,^ ^b^ ^,^ ^d^⊕○○○
NP	Very serious ↓↓	Not serious	Serious ↓	Not serious	4.6 to 4.9 (3.7–6.2)	Very low^a^ ^,^ ^b^ ^,^ ^c^⊕○○○
Knee OA	Very serious ↓↓	Not serious	Serious ↓	Not serious	6.0 to 7.9 (5.2–8.9)	Very low ⊕○○○

^a^ Downgraded −1 for risk of bias, due to concerns about the credibility of the model, influenced by its conceptualisation and structure.

^b^ Downgraded −1 for risk of bias, due to concerns about the certainty and exhaustiveness of model inputs.

^c^ Downgraded −1 for indirectness, due to indirectness of model inputs with respect to the model, influenced by lack of primary data input studies.

^d^ Downgraded −1 for inconsistency, due to unexplained variability in modelled outputs.

## Discussion

### Statement of Findings

The analysed GBD 2019 primary data input studies for LBP, NP, and knee OA in our meta-epidemiological study had methodological shortcomings and had low coverage for the studied period. Similarly, the certainty of modelled prevalence estimates of LBP, NP, and knee OA in Australia, Brazil, Canada, Spain, and Switzerland ranged between very low and low.

### Meaning and Importance of Findings

Primary data input studies had limitations mainly related to their representativeness, case definitions, and validity of their instruments to measure prevalence. Although we used an inclusive prevalence period recall of up to 1 year as acceptable in assessing case definitions, recall periods of more than 4 weeks might be prone to memory bias [101]. Furthermore, between 1990 and 2019 there were a substantial number of years without any primary study coverage, which might compromise temporal interpolations of modelled estimates. Our findings indicate that GBD modelled prevalence estimates should be interpreted with caution and acknowledge inherent weaknesses of primary input studies and sources of uncertainty of the modelling process. Our approach also conceivably supports the feasibility of establishing methodological approaches to rate the certainty of GBD metrics.

An interesting result to emerge from the GRADE application, was the observed consistency and precision of modelled prevalence estimates. The direction and width of modelled prevalence uncertainty intervals were not sensitive to the number of primary input studies for a given country. This observation, which is in agreement with a recent correspondence to a GBD analysis in Iran [102, 103], potentially highlights that modelling assumptions, country-specific covariates, and alternative primary data may also influence modelled estimates. Major advances are occurring in terms of GBD best reporting practice and Burden of Proof methodology [12, 104]. Unlike our proposed GRADE approach for certainty of modelled prevalence estimates, Burden of Proof focuses on risk-outcome relationships, aggregates evidence across multiple studies, and enables a quantitative comparison of risk-outcome pairs, ultimately assessing the strength of evidence with an intuitive star rating. Future results of evaluations of GBD modelling performance, sensitivity analyses, and star ratings to summarise evidence beyond risk-outcome pairs will continue to advance this body of knowledge.

In our study, two exceptions to the consistency of GBD modelled estimates were observed (Figure 2). First, LBP modelled prevalence trends in Switzerland between 2016 and 2019, decreased from 19.1% (95% UI, 18.0%–20.2%) to 15.0% (13.1%–17.2%), a change that was not observed in other countries. Second, NP modelled prevalence trends in Spain between 2001 and 2014 decreased from 3.5% (95% UI: 2.8%–4.3%) in 2001 to 2.6% (2.2%–3.3%) in 2005 and then increased to 3.7% (2.9%–4.6%) in 2014, creating a U-shape trend line. Importantly, these variations in modelled prevalence estimates could not be unambiguously explained by any of the primary data input studies (primary input studies for Switzerland, 2016–2019: 0 studies; primary data input studies for Spain, 2001–2014: 1 study).

### Our Findings in the Context of Existing Evidence

Our results expand on those reported by Tamrakar et al., who explored primary data input studies for LBP in GBD 2017 [11], examining more countries and using an alternative approach to rate the completeness and quality of primary input studies. Brhlikova et al. also assessed the quality of primary studies for depression in GBD 2000, pointing out their limitations [105]. Our study adds to the existing body of knowledge by applying a novel GRADE approach to rate the certainty of GBD modelled prevalence estimates, aiming to improve the assessment and communication of uncertainty. This addition is relevant for GBD, as there are national burden of disease initiatives that have developed certainty frameworks based on the availability and transformation applied to primary data [106, 107].

### Alternative Explanation and Challenges of GRADE

Despite our analysis, primary data input studies were not the unique source of input for GBD 2019. Access and evaluation of opportunistic surveys and claims data may have resulted in alternative findings. Additionally, we did not have enough information to fully assess all dimensions of credibility of the model, to judge the input parameters to which modelled prevalence estimates were sensitive, nor to assess the potential impact of studies that were excluded from the GBD 2019 systematic reviews. We also encountered challenges in the application of GRADE Guidelines 30—although options to incorporate multiple model outputs in decision-making are available, we used an existing model without adaptation due to resource limitations. Adapting the GBD model would have required all input data relevant for prevalence estimation, GBD modelling expertise, and extensive computational resources.

### Strengths and Limitations

The abundance of secondary analyses of GBD MSK data contrasts with a paucity of methodological publications [108–110], and a scarcity of analytical approaches to facilitate the interpretation of modelled estimates. Among the strengths of our study, the use of a validated tool to assess risk of bias of primary input studies and a well-established framework to assess the certainty of modelled evidence should be highlighted. In addition, choosing prevalence estimates could also be considered a strength, since these are not affected by disability weights, severity distributions, or comorbidity corrections, requiring fewer dimensions to judge their certainty. The exclusion of disability weight-based metrics adds pragmatism since there are potential biases in weighted average disability weights created using worldwide factors [111], and inter-country variations in the subjective valuation of health states are expected [108].

Our study has several limitations. First, we purposively explored a select number of MSK conditions and countries. That said, GBD primary data have better coverage and lower risk of bias for countries with higher incomes [11], suggesting that our risk of bias and certainty findings may approximate a best case scenario. Second, we restricted our analysis to primary data input studies identifiable in the GBD 2019 Data Input Sources Input Tool and did not include other sources of GBD input data. Third, we did not analyse neighbouring countries, even though GBD uses them for modelling estimation. Fourth, we excluded the category of “other MSK disorders”, despite its high modelled prevalence, due to its heterogeneity. Fifth, although some studies for a newer GBD have been published [112], we did not have access to up-to-date primary input studies. Sixth, the validity of GRADE for assessing the certainty of modelled GBD prevalence estimates is unknown and other conceptual frameworks may be more suitable [113]. The possibility that alternative assessment approaches would have yielded different certainty findings cannot be ruled out.

### Suggestions for Future Research

Along with progress in the estimation of prevalence in GBD, it is crucial to encourage high-quality primary data on risk factors and advance methods to obtain accurate, time-varying MSK severity distributions by triangulation of data sources across locations [114]. This importance is accentuated given the marked contribution of MSK disorders to YLD, their wide range of severity weights across health states, and the plausible exacerbation of MSK severity within the context of the COVID pandemic. An important issue to strengthen population-based MSK research is to promote the use of optimal case definitions (including activity limiting considerations, explicit anatomical location, duration, and appropriate prevalence periods of maximum 4 weeks to minimize memory bias) [101], as well as the use of validated tools to measure prevalence. Collaborative networks, such as the European Burden of Disease Network, may be a promising driver of improvement in supporting methodological advances [115]. Additional studies may elucidate the optimal approach to rate the certainty of GBD estimates. Advancement in this area could help GBD end-users, some of whom lack technical ability to comprehend complex models and may be pressured to make policy decisions. The combination of the GBD premise that “providing estimates with corresponding uncertainty is better than not providing any estimate at all” [116], and the suggested poor legitimization of MSK disorders at times [117], calls for crucial advances in understanding the true burden of MSK conditions.

### Conclusion

The primary data input studies that underpinned the GBD 2019 prevalence estimates of LBP, NP, and knee OA in Australia, Brazil, Canada, Spain, and Switzerland had methodological shortcomings. This meta-epidemiological work also suggests that the certainty of GBD modelled prevalence estimates for these three MSK pain conditions is limited mainly due to risk of bias and indirectness. Future primary input studies with lower risk of bias, and the optimal assessment of uncertainty in modelled outputs, will likely improve the certainty of modelled prevalence estimates.

## Data Availability

Datasets and primary data input studies are publicly available. These data can be found at: https://ghdx.healthdata.org/gbd-2019/data-input-sources, https://vizhub.healthdata.org/gbd-results/.
