# Supplementary material for: Certainty of the Global Burden of Disease 2019 Modelled Prevalence Estimates for Musculoskeletal Conditions: A Meta-Epidemiological Study
Source: Int J Public Health. 2023 May 31;68:1605763. doi: 10.3389/ijph.2023.1605763 (PMC10266422; doi:10.3389/ijph.2023.1605763)
Supplement: Supplementary file 1 [file DataSheet1.docx]

Muñoz Laguna J, Puhan MA, Rodríguez Artalejo F, De Pauw R, Wyper GMA, Devleesschauwer B, Santos JV and Hincapié CA (2023). Certainty of the Global Burden of Disease 2019 Modelled Prevalence Estimates for Musculoskeletal Conditions: A Meta-Epidemiological Study. Int J Public Health 68:1605763. doi: 10.3389/ijph.2023.1605763

**Supplementary Datasheet S1**

**Table of contents**

Identification of primary data input studies and GBD 2019 modelled estimates. 3

Applications and requests to access data. 3

Statistical Appendix. Standard error considerations for prevalence estimates derived from primary data input studies. 4

Risk of bias tool. Hoy’s risk of bias tool for population-based prevalence studies. 5

sTable 1. Number of low back pain, neck pain, and knee osteoarthritis primary data input studies from 1990 to 2019, informing GBD 2019 modelled epidemiologic estimates for five countries. 9

sFigure 1. Choropleth map representing the number of low back pain primary data input studies from 1990 to 2019, informing GBD 2019 modelled epidemiologic estimates for five countries 10

sFigure 2. Choropleth map representing the number of neck pain primary data input studies from 1990 to 2019, informing GBD 2019 modelled epidemiologic estimates for five countries. 11

sFigure 3. Choropleth map representing the number of knee osteoarthritis primary data input studies from 1990 to 2019, informing GBD 2019 modelled epidemiologic estimates for five countries. 12

sTable 2. Detailed evidence table of primary data input studies informing GBD 2019 modelled epidemiologic estimates of low back pain in Australia, Brazil, Canada, Spain, and Switzerland. 13

sTable 3. Detailed evidence table of primary data input studies informing GBD 2019 modelled epidemiologic estimates of neck pain in Australia, Brazil, Canada, Spain, and Switzerland. 33

sTable 4. Detailed evidence table of primary data input studies informing GBD 2019 modelled epidemiologic estimates of knee osteoarthritis in Australia, Brazil, Canada, Spain, and Switzerland. 34

sFigure 4. Traffic-light plot representing the risk of bias assessment of primary data input studies informing GBD 2019 modelled prevalence estimates of low back pain, neck pain, and knee osteoarthritis in Australia, Brazil, Canada, Spain, and Switzerland. 35

sFigure 5. Summary plot representing the risk of bias assessment of primary data input studies informing GBD 2019 modelled prevalence estimates of low back pain, neck pain, and knee osteoarthritis in Australia, Brazil, Canada, Spain, and Switzerland. 42

sTable 5. GBD 2019 modelled prevalence estimates for low back pain in Australia, Brazil, Canada, Spain, and Switzerland from 1990 to 2019. 43

sTable 6. GBD 2019 modelled prevalence estimates for neck pain in Australia, Brazil, Canada, Spain, and Switzerland from 1990 to 2019. 45

sTable 7. GBD 2019 modelled prevalence estimates for knee osteoarthritis in Australia, Brazil, Canada, Spain, and Switzerland from 1990 to 2019. 47

sTable 8. Certainty assessment of the GBD 2019 modelled prevalence estimates of low back pain in Australia, Brazil, Canada, Spain, and Switzerland (1990 to 2019) following GRADE 30 Guidelines. . 49

sTable 9. Certainty assessment of the GBD 2019 modelled prevalence estimates of neck pain in Australia, Brazil, Canada, Spain, and Switzerland (1990 to 2019) following GRADE 30 Guidelines. 51

sTable 10. Certainty assessment of the GBD 2019 modelled prevalence estimates of knee osteoarthritis in Australia, Brazil, Canada, Spain, and Switzerland (1990 to 2019) following GRADE 30 Guidelines. 53

**References for Supplementary Datasheet S1** …..…………………………………………………………………………………………………….. 55

**Identification of primary data input studies and GBD 2019 modelled prevalence estimates.**

*Identifying primary data input studies*

1. Access <https://ghdx.healthdata.org/gbd-2019/data-input-sources>

2. Introduce the following search terms: Components = “Nonfatal Health Outcomes”; Causes = “Low back pain” OR “Neck pain” OR “Osteoarthritis knee”; Impairments = NA; Locations = “Australia” OR “Brazil” OR “Canada” OR “Spain” OR “Switzerland”

3. Click on “download source metadata CSV” and “download citations CSV”

*Identifying GBD 2019 modelled prevalence estimates*

1. Access <https://vizhub.healthdata.org/gbd-results/>

2. Introduce the following search terms: GBD Estimate = “Cause of death or injury”; Measure = “Prevalence”; Metric = “Percent”; Cause = “Low back pain” OR “Neck pain” OR “Osteoarthritis knee”; Location = “Australia” OR “Brazil” OR “Canada” OR “Spain” OR “Switzerland”; Age = “All ages”; Sex = “Both”; Year = “Select all”

3. Click on “Search” and “Download”

**Applications and requests to access data.**

The applications to access the Swiss Household Panel Survey data and the World Health Surveys were filled out by the lead researcher.

- SWISSUbase contract reference: 102572 (accepted on May 20^th^, 2022); Reference study: 6097; Reference dataset: 932
- World Health Survey multi study request: ID number 4024; Status: Approved (accepted on May 1^st^, 2022)

**Statistical Appendix.** Standard error considerations for prevalence estimates derived from primary data input studies.

When the standard error for a prevalence estimate was not provided, we followed the approach suggested by Hoy [1] to estimate it (Wald approach accounting for the design effect of the study):

$$SE=\sqrt{\frac{DE\cdot p\cdot\left( 1-p \right)}{N}}$$

where:

- $DE$ is the design effect (DE = 1 if the study used a simple or stratified sampling technique; DE = 2 if the study used a cluster sampling and/or did not report a design effect)
- $p$ is the prevalence estimate
- $N$ is the sample size

The equivalent {base} R code could go as follows:

*# Helper function to estimate a standard error and obtain the lower and upper limits of a 95% confidence interval for a prevalence estimate as output*

*se <- function(DE, p, N) {*

*se <- sqrt((DE * p * (1-p))/N)*

*ci_low <- p - 1.96 * se*

*ci_high <- p + 1.96 * se*

*out <- list(ci_low, ci_high)*

*return(out)*

*}*

When relative standard errors (RSE) of proportions were provided (standard error expressed as a percentage of the estimate to which it relates), the standard errors were derived:

$$RSE\%=\frac{SE}{p}\left( 100 \right)$$

$$SE=(RSE\%* p)/100$$

Where:

- $p$ refers to the prevalence estimate
- $RSE\%$ represents the relative standard error expressed as a percentage

**Risk of bias tool.** Hoy’s risk of bias tool for population-based prevalence studies.

Hoy’s tool [2] is designed to assess the risk of bias in population-based prevalence studies. When there is insufficient information in an article to permit a judgement for a particular item, the instrument encourages researchers to answer “No (HIGH RISK)” for that item.

| Risk of bias item | Criteria for answers (please circle one option) | Additional notes and examples |
| --- | --- | --- |
| *External Validity* | | |
| 1. Was the study’s target population a close representation of the national population in relation to relevant variables, e.g. age, sex, occupation? | - Yes (LOW RISK): The study’s target population was a close representation of the national population. - No (HIGH RISK): The study’s target population was clearly NOT representative of the national population. | The target population refers to the group of people or entities to which the results of the study will be generalised. Examples:  The study was a national health survey of people 15 years and over and the sample was drawn from a list that included all individuals in the population aged 15 years and over. The answer is: Yes (LOW RISK).  The study was conducted in one province only, and it is not clear if this was representative of the national population. The answer is: No (HIGH RISK).  The study was undertaken in one village only and it is clear this was not representative of the national population. The answer is: No (HIGH RISK). |
| 2. Was the sampling frame a true or close representation of the target population? | - Yes (LOW RISK): The sampling frame was a true or close representation of the target population. - No (HIGH RISK): The sampling frame was NOT a true or close representation of the target population. | The sampling frame is a list of the sampling units in the target population and the study sample is drawn from this list. Examples:  The sampling frame was a list of almost every individual within the target population. The answer is: Yes (LOW RISK).  The cluster sampling method was used and the sample of clusters/villages was drawn from a list of all villages in the target population. The answer is: Yes (LOW RISK).  The sampling frame was a list of just one particular ethnic group within the overall target population, which comprised many groups. The answer is: No (HIGH RISK). |
| 3. Was some form of random selection used to select the sample, OR, was a census undertaken? | - Yes (LOW RISK): A census was undertaken, OR, some form of random selection was used to select the sample (e.g. simple random sampling, stratified random sampling, cluster sampling, systematic sampling). - No (HIGH RISK): A census was NOT undertaken, AND some   form of random selection was NOT used to select the sample. | A census collects information from every unit in the sampling frame. In a survey, only part of the sampling frame is sampled. In these instances, random selection of the sample helps minimise study bias. Examples:  The sample was selected using simple random sampling. The answer is: Yes (LOW RISK).  The target population was the village and every person in the village was sampled. The answer is: Yes (LOW RISK).  The nearest villages to the capital city were selected in order to save on the cost of fuel. The answer is: No (HIGH RISK). |
| 4. Was the likelihood of non-response bias minimal? | - Yes (LOW RISK): The response rate for the study was >/=75%, OR, an analysis was performed that showed no significant difference in relevant demographic characteristics between responders and non- responders - No (HIGH RISK): The response rate was <75%, and if any analysis comparing responders and non-responders was done, it showed a significant difference in relevant demographic characteristics between responders and non-responders. | Examples:  The response rate was 68%; however, the researchers did an analysis and found no significant difference between responders and non-responders in terms of age, sex, occupation and socioeconomic status. The answer is: Yes (LOW RISK).  The response rate was 65% and the researchers did NOT carry out an analysis to compare relevant demographic characteristics between responders and non-responders. The answer is: No (HIGH RISK).  The response rate was 69% and the researchers did an analysis and found a significant difference in age, sex and socio-economic status between responders and non-responders. The answer is: No (HIGH RISK). |
| *Internal Validity* | | |
| 5. Were data collected directly from the subjects (as opposed to a proxy)? | - Yes (LOW RISK): All data were collected directly from the subjects. - No (HIGH RISK): In some instances, data were collected from a proxy. | A proxy is a representative of the subject. Examples:  All eligible subjects in the household were interviewed separately. The answer is: Yes (LOW RISK).  A representative of the household was interviewed and questioned about the presence of low back pain in each household member. The answer is: No (HIGH RISK). |
| 6. Was an acceptable case definition used in the study? | - Yes (LOW RISK): An acceptable case definition was used. - No (HIGH RISK): An acceptable case definition was NOT used. | For a study on low back pain, the following case definition was used: “Low back pain is defined as activity-limiting pain lasting more than one day in the area on the posterior aspect of the body from the bottom of the 12th rib to the lower gluteal folds.” The answer is: Yes (LOW RISK).  For a study on back pain, there was no description of the specific anatomical location “back” referred to. The answer is: No (HIGH RISK).  For a study on osteoarthritis, the following case definition was used: “Symptomatic osteoarthritis of the hip or knee, radiologically confirmed as Kellgren-Lawrence grade 2-4”. The answer is: LOW RISK. |
| 7. Was the study instrument that measured the parameter of interest (e.g. prevalence of low back pain) shown to have reliability and validity (if necessary)? | - Yes (LOW RISK): The study instrument had been shown to have reliability and validity (if this was necessary), e.g. test-re- test, piloting, validation in a previous study, etc. - No (HIGH RISK): The study instrument had NOT been shown to have reliability or validity (if this was necessary). | The authors used the COPCORD questionnaire, which had previously been validated. They also tested the inter-rater reliability of the questionnaire. The answer is: Yes (LOW RISK).  The authors developed their own questionnaire and did not test this for validity or reliability. The answer is: No (HIGH RISK). |
| 8. Was the same mode of data collection used for all subjects? | - Yes (LOW RISK): The same mode of data collection was used for all subjects. - No (HIGH RISK): The same mode of data collection was NOT used for all subjects. | The mode of data collection is the method used for collecting information from the subjects. The most common modes are face-to-face interviews, telephone interviews and self-administered questionnaires. Examples:  All eligible subjects had a face-to-face interview. The answer is: Yes (LOW RISK).  Some subjects were interviewed over the telephone, and some filled in postal questionnaires. The answer is: No (HIGH RISK). |
| 9. Was the length of the shortest prevalence period for the parameter of interest appropriate? | - Yes (LOW RISK): The shortest prevalence period for the parameter of interest was appropriate (e.g. point prevalence, one-week prevalence, one-year prevalence). - No (HIGH RISK): The shortest prevalence period for the parameter of interest was not appropriate (e.g. lifetime   prevalence) | The prevalence period is the period that the subject is asked about e.g. “Have you experienced low back pain over the previous year?” In this example, the prevalence period is one year. The longer the prevalence period, the greater the likelihood of the subject forgetting if they experienced the symptom of interest (e.g. low back pain). Examples:  Subjects were asked about pain over the past week. The answer is: Yes (LOW RISK).  Subjects were only asked about pain over the past three years. The answer is: No (HIGH RISK). |
| 10. Were the  numerator(s) and denominator(s) for the parameter of interest appropriate? | - Yes (LOW RISK): The paper presented appropriate numerator(s) AND denominator(s) for the parameter of interest (e.g. the prevalence of low back pain). - No (HIGH RISK): The paper did present numerator(s) AND denominator(s) for the parameter of interest but one or more of these were inappropriate. | There may be errors in the calculation and/or reporting of the numerator and/or denominator. Examples:  There were no errors in the reporting of the numerator(s) AND denominator(s) for the prevalence of low back pain. The answer is: Yes (LOW RISK).  In reporting the overall prevalence of low back pain (in both men and women), the authors accidentally used the population of women as the denominator rather than the combined population. The answer is: No (HIGH RISK). |
| 11. Summary item on the overall risk of study bias | | |
| LOW RISK OF BIAS: Further research is very unlikely to change our confidence in the estimate.  MODERATE RISK OF BIAS: Further research is likely to have an important impact on our confidence in the estimate and may change the estimate.  HIGH RISK OF BIAS: Further research is very likely to have an important impact on our confidence in the estimate and is likely to change the estimate | | |

| Condition  Country | N | Source metadata rows | Link |
| --- | --- | --- | --- |
| Low back pain | | |  |
| Australia | 12^a^ | 247 | <https://ghdx.healthdata.org/gbd-2019/data-input-sources?components=5&causes=630&locations=71> |
| Brazil | 10 | 101 | <https://ghdx.healthdata.org/gbd-2019/data-input-sources?components=5&causes=630&locations=135> |
| Canada | 7 | 66 | <https://ghdx.healthdata.org/gbd-2019/data-input-sources?components=5&causes=630&locations=101> |
| Spain | 19 | 242 | <https://ghdx.healthdata.org/gbd-2019/data-input-sources?components=5&causes=630&locations=92> |
| Switzerland | 19^b^ | 464 | <https://ghdx.healthdata.org/gbd-2019/data-input-sources?components=5&causes=630&locations=94> |
| Global | 455 | 24,834 | <https://ghdx.healthdata.org/gbd-2019/data-input-sources?components=5&causes=630&locations=1> |
| Neck pain | | |  |
| Australia | 0 | 0 | <https://ghdx.healthdata.org/gbd-2019/data-input-sources?components=5&causes=631&locations=71> |
| Brazil | 1 | 32 | <https://ghdx.healthdata.org/gbd-2019/data-input-sources?components=5&causes=631&locations=135> |
| Canada | 0 | 0 | <https://ghdx.healthdata.org/gbd-2019/data-input-sources?components=5&causes=631&locations=101> |
| Spain | 1 | 34 | <https://ghdx.healthdata.org/gbd-2019/data-input-sources?components=5&causes=631&locations=92> |
| Switzerland | 0 | 0 | <https://ghdx.healthdata.org/gbd-2019/data-input-sources?components=5&causes=631&locations=94> |
| Global | 84 | 17,752 | <https://ghdx.healthdata.org/gbd-2019/data-input-sources?components=5&causes=631&locations=1> |
| Knee osteoarthritis | | |  |
| Australia | 0 | 0 | <https://ghdx.healthdata.org/gbd-2019/data-input-sources?components=5&causes=1015&locations=71> |
| Brazil | 0 | 0 | <https://ghdx.healthdata.org/gbd-2019/data-input-sources?components=5&causes=1015&locations=135> |
| Canada | 1 | 28 | <https://ghdx.healthdata.org/gbd-2019/data-input-sources?components=5&causes=1015&locations=101> |
| Spain | 2 | 26 | <https://ghdx.healthdata.org/gbd-2019/data-input-sources?components=5&causes=1015&locations=92> |
| Switzerland | 0 | 0 | <https://ghdx.healthdata.org/gbd-2019/data-input-sources?components=5&causes=1015&locations=94> |
| Global | 67 | 9,757 | <https://ghdx.healthdata.org/gbd-2019/data-input-sources?components=5&causes=1015&locations=1> |
| ^a^ HBSC 2010 for Austria was misclassified.[3]  ^b^ Balagué et al. 1994 provided information for Switzerland for the year 1989.[4] | | | |

**sTable 1.** Number of low back pain, neck pain, and knee osteoarthritis primary data input studies from 1990 to 2019, informing GBD 2019 modelled epidemiologic estimates for five countries.

**sFigure 1.** Choropleth map representing the number of low back pain primary data input studies from 1990 to 2019, informing GBD 2019 modelled epidemiologic estimates for five countries (countries not included in the analysis are shown in gray; created with Datawrapper, [**https://app.datawrapper.de/**](https://app.datawrapper.de/)).


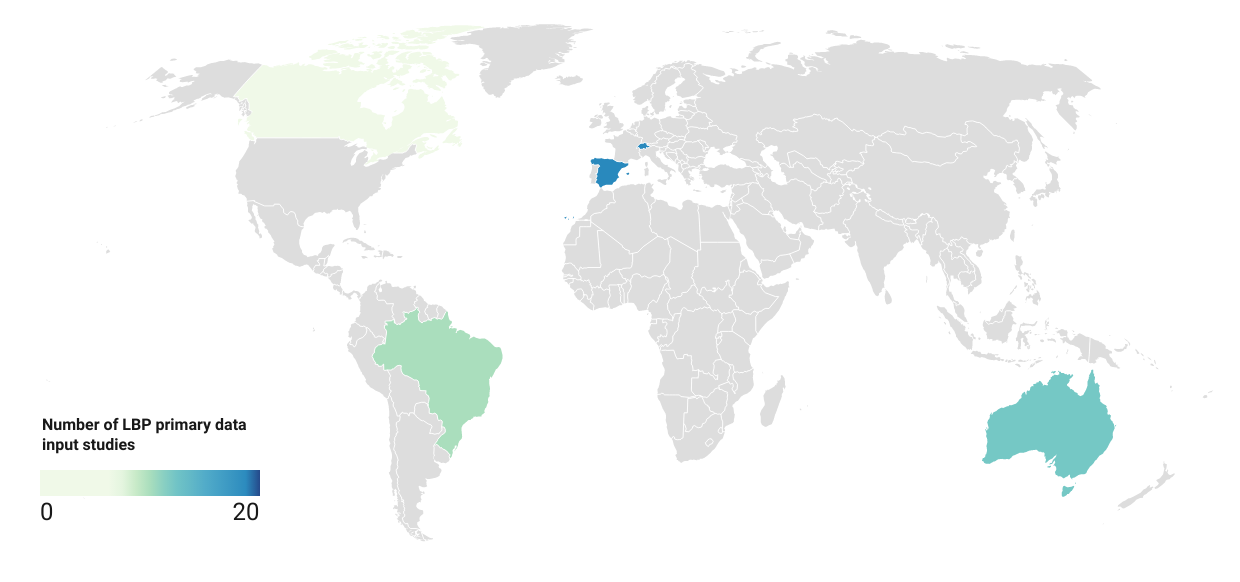


**sFigure 2.** Choropleth map representing the number of neck pain primary data input studies from 1990 to 2019, informing GBD 2019 modelled epidemiologic estimates for five countries (countries not included in the analysis are shown in gray; created with Datawrapper, [**https://app.datawrapper.de/**](https://app.datawrapper.de/)).


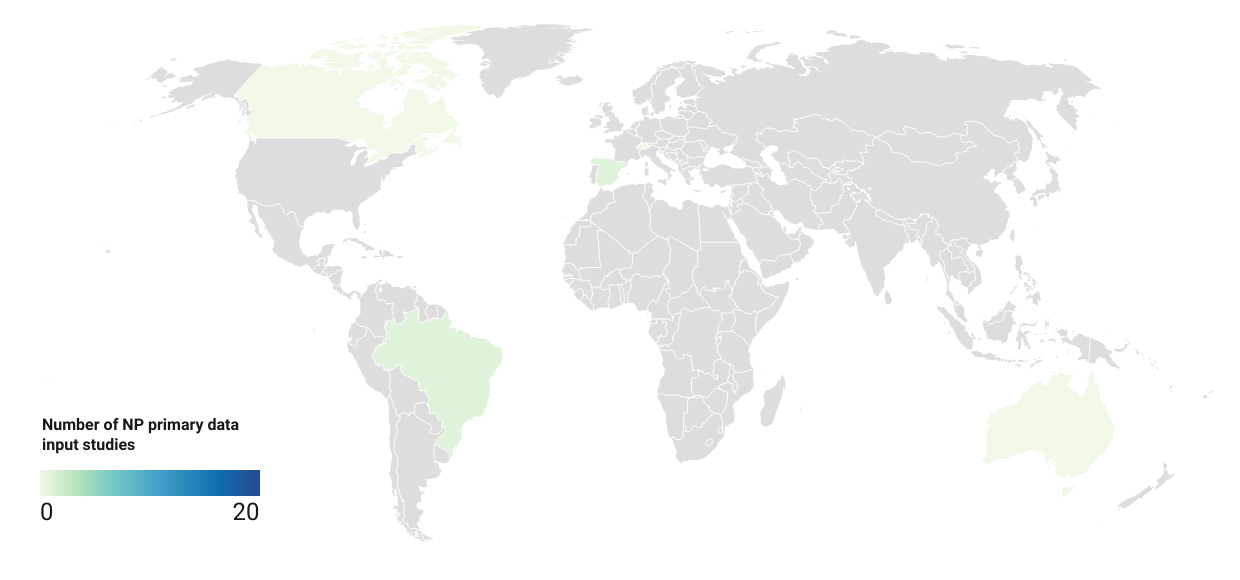


**sFigure 3.** Choropleth map representing the number of knee osteoarthritis primary data input studies from 1990 to 2019, informing GBD 2019 modelled epidemiologic estimates for five countries (countries not included in the analysis are shown in gray; created with Datawrapper, [**https://app.datawrapper.de/**](https://app.datawrapper.de/)).


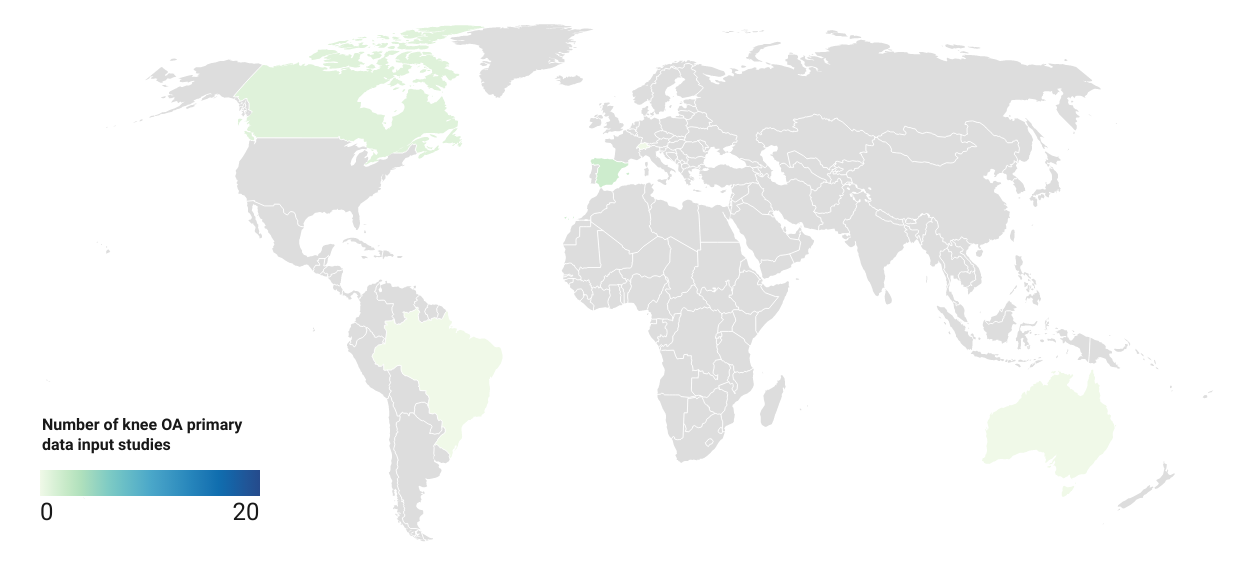


**sTable 2.** Detailed evidence table of primary data input studies informing GBD 2019 modelled epidemiologic estimates of low back pain in Australia, Brazil, Canada, Spain, and Switzerland.

| Study, year | Sampling frame | Sampling design  (Sample size, N) | Response (%) | Years covered |  | Ages (y)  Women (%) |  | Case definition | Prevalence estimate % (95% CI) |
| --- | --- | --- | --- | --- | --- | --- | --- | --- | --- |
| Australia | | | | | | | | | |
| NHS, 1995 [5,6] | National | Stratified multistage  [N= 21,787 households (fully or partly responding);  N = 53,828 people  (unweighted)] | 92% (Household);  97% (Individual) | 1995 |  | 0-75+  51% |  | Long-term back problems (unspecified) were defined as current back problems that had lasted or were expected to last 6 months or more; sciatica, intervertebral disc disorders, and spine curvature were not included in this category | 17.6 (17.0 to 18.2) |
| NHS, 2001 [7,8] | National | Stratified multistage  [N = 17,918 households (fully responding);  N = 26,863 persons  (unweighted)] | 92% (General, Household);  89% (Indigenous,  Household);  NA% (Individual) | 2001 |  | 0-75+  53% |  | Long-term back problems (unspecified) were defined as current back problems that had lasted or were expected to last 6 months or more; sciatica, intervertebral disc disorders, and spine curvature were included in this category | 21.0 (20.4 to 21.6)^a^ |
| SDAC, 2003 [9,10] | National | Multistage  [N = 36,241 people^b^ (Household);  N = 5,145 people^c^  (Cared-accommodation)] | 89% (Household);  92% (Cared-accommodation) | 2003 |  | 0-85+  53% |  | SDAC first determines whether there are people in a household who currently have conditions that have lasted or are likely to last at least 6 months. For this case definition, “back problems” were the main condition causing disability. Disabling and recurrent pain had to be experienced some time in the last twelve months | 3.1 (2.9 to 3.3) |
| WHS Australia, 2003 [11] | National | Multistage cluster  [(N = 1,727  (Household);  N = 1,846  (Individual)] | NA | 2003 |  | 15-80+ |  | Back pain (including disc problems) in the last 30 days: “Have you experienced back pain (including disc problems) during the last 30 days?” | DC: 44.1 (40.8 to 47.3); CATI: 39.0 (35.8 to 42.2); Combined: 41.6 (39.3 to 43.9)^h^ |
| Walker, 2004 [12] | National | Stratified random  (N = 1,913) | 69% | 2001 |  | 18-70+  53% |  | A diagram of a mannequin defined the low back as a shaded area between the last ribs and the gluteal folds. Direct questions (i.e., ‘‘Have you had low back pain in the past...’’) were asked about cumulative lifetime prevalence, point, and other prevalence periods | Point (age and gender standardized): 25.6  (23.7 to 27.6); One day: 31.7 (29.7 to 33.9); Two weeks: 45.0 (42.8 to 47.3);  One month: 52.2 (50.0 to 54.5); Six months (age and gender standardized): 65.0 (62.8 to 67.1);^e^ Twelve months: 67.6 (65.5 to 69.7);  Lifetime (age and gender standardized): 78.9 (77.0 to 80.7) |
| NHS, 2004-2005 [13,14] | National | Stratified multistage  [N = 19,501 households  (fully responding);  N = 25,906 persons  (Individual, unweighted)] | 89% (Household);  NA% (Individual) | 2004-2005 |  | 0-75+  53% |  | Long-term back problems (unspecified) were defined as current back problems that had lasted or were expected to last 6 months or more; sciatica, intervertebral disc disorders, and spine curvature were included in this category | 16.4 (15.8 to 17.0)^a^ |
| Grimmer, 2006 [15] | Subnational (Adelaide) | Random cluster  (N = 434) | 83%  (baseline response) | 1999-2003 |  | 13-17  43% |  | Body pain in the last week: “Have you had any type of pain in the last week? If yes, circle the area(s) (on the body chart) where you get the pain(s)” | Year 1999: 7.1 (3.7 to 10.5); Year 2000: 12.7 (7.5 to 17.9); Year 2001: 15.3 (9.5 to 21.1); Year 2002: 17.3; (10.6 to 24.0)  Year 2003: 16.7 (8.9 to 24.5) |
| NHS, 2007-2008 [16,17] | National | Stratified multistage  (N = 15,792  (Fully responding Households);  N = 20,788 persons  (Individual, unweighted)) | 87% (Household);  NA% (Individual) | 2007-2008 |  | 0-75+  52% |  | Long-term back problems (unspecified) were defined as current back problems that had lasted or were expected to last 6 months or more; sciatica, intervertebral disc disorders, and spine curvature were included in this category | 14.5 (13.7 to 15.3)^a^ |
| SDAC, 2009 [18,19] | National | Multistage  [N =64,213 people^b^  (Household);  N = 9,470 people^c^  (Cared-accommodation)] | 90% (Household);  91% (Cared-accommodation) | 2009 |  | 0-85+  53% |  | SDAC first determines whether there are people in a household who currently have conditions that have lasted or are likely to last at least 6 months. For this case definition, “back problems” were the main condition causing disability. Disabling and recurrent pain had to be experienced some time in the last twelve months | 2.9 (2.8 to 3.0) |
| HBSC Austria, 2010 [3] | National | Multistage clustered [N = 6,493 (national report);  N = 5,003 (respondents, international report)] | 72% | 2010 |  | 10-19  52% |  | Frequency of low back pain in the last six months: “In the last six months, how often have you had back pain?” Those who responded “almost every month” or more frequently were considered cases | 37.1 (35.4 to 38.8) |
| Broom, 2012 [20] | National | Random  (N = 9,820) | 84% | 2004-2007 |  | 18-75  100% |  | Care-seeking episode of back pain in the previous twelve months | 54.8 (53.8 to 55.8) |
| O'Sullivan, 2012 [21] | Subnational (Perth) | Unspecified  (N = 1,288) | 46% of the original sample completed some aspect of follow-up; 89% of those who completed some aspect of follow-up, completed the paper  questionnaire covering low back pain prevalence | 2008 |  | 16-17  53% |  | The experience of low back pain was determined using the Nordic low back pain questionnaire, with modifications: “Has your low back been painful at any time in the last month?”^e^ | Current non-chronic: 12.3 (9.8 to 14.8); Current chronic: 19.9 (16.8 23.0) |
| Brazil |  |  |  |  |  |  |  |  |  |
| WHS Brazil, 2003 [22] | National | Multistage cluster  [N = 5,000 (Household);  N = 4,999 (Individual)] | 70% (Household);  100% (Individual) | 2003 |  | 15-80+  52% |  | Back pain (including disc problems) in the last 30 days: “Have you experienced back pain (including disc problems) during the last 30 days?” | 52.6 (50.6 to 54.6)^d^ |
| Silva, 2004 [23] | Subnational (Pelotas) | Multistage, combination of cluster and systematic  (N = 3,182) | 93% | 2002 |  | 20-70+  57% |  | The presence of chronic low back pain was established by two criteria: 1) identification of the lumbar region as the site of pain in a figure and 2) presence of pain for seven weeks or more | 4.2 (3.5 to 4.9) |
| Mendoza-Sassi, 2006 [24] | Subnational  (Rio Grande) | Multistage, combination of cluster, simple, and systematic  (N = 1,259) | 93% | 2000 |  | 15-94  54% |  | The presence of signs and symptoms of low back pain in the previous two months (self-reported) was identified from a list of 18 items, built from previous studies^f^ | 35.1 (32.5 to 37.7) |
| Blay, 2007 [25] | Subnational  (Rio Grande do Sul) | Multistage stratified random  (N = 6,961) | 99% | 2004 |  | 60-81+  66% |  | Chronic low back pain was self-reported as back pain in treatment in the last six months: “In the last six months have you had back ache in treatment?” | Women: 47.5 (47.5 to 47.5); Men: 34.7 (34.7 to 34.7); Combined: 43.1 (41.9 to 44.3) |
| De Vitta, 2011 [26] | Subnational (Bauru) | Unspecified  (N = 1,236) | 93% | 2007 |  | 11-14  52% |  | Low back pain was measured with the Nordic questionnaire, adapted to the Brazilian culture: Low back pain (as shown in an image) was defined as pain or discomfort not related to trauma or menstrual pain in the last 12 months | 19.5 (17.3 to 21.7) |
| Ferreira, 2011 [27] | Subnational (Pelotas) | Multistage cluster  (N = 972) | 91% | 2007 |  | 20-69  57% |  | The question used to define the outcome of spinal pain was: “In the last year, have you had any pain or discomfort in the spine somewhere (as shown in a colored human picture)?”^g^ | 40.0 (36.9 to 43.2) |
| Onofrio, 2012 [28] | Subnational (Pelotas) | Stratified random  (N = 1,233) | 91% | 2009 |  | 13-19  54% |  | 30-day low back pain recall: 1) “Have you ever had low back pain in the site shown in this figure (shaded diagram)?” 2) “When did you have low back pain (maximum 30 days of recall)?” | 13.7 (11.8 to 15.6) |
| Meziat Filho, 2015 [29] | Subnational  (Rio de Janeiro) | Unspecified  (N = 1,102) | Unspecified | 2012-2013 |  | 14-20  53% |  | 1) “Has your low back (as shown on a body diagram) been painful at any time in the previous month?”, and 2) “Has your low back pain ever lasted for more than 3 months?”^h^ | Acute: 28.6 (25.9 to 31.2); Chronic: 18.2 (15.9 to 20.5) |
| Depintor, 2016 [30] | Subnational  (São Paulo) | Random probabilistic  (N = 826) | 75% | 2011-2012 |  | 15-60+  69% |  | Chronic low back pain (persistent pain for three or more months) was defined as pain located in the region bounded by the twelfth rib, superiorly; the gluteal line, inferiorly; and the anterior axillary line, anteriorly^j^ | 18.4 (15.8 to 21.2) |
| Noll, 2016 [31] | Subnational (Teutonia) | Unspecified  (N = 1,597) | 93% | 2013-2014 |  | 11-16  46% |  | Back pain in the last three months: “Have you felt (or have been feeling) back pain in the last three months?”^i^ | 55.7 (53.1 to 58.3) |
| Canada |  |  |  |  |  |  |  |  |  |
| Liira, 1996 [32] | Subnational (Ontario) | Unspecified  (N = 38,540) | 88% | 1990 |  | 16-64  50% |  | Long-term and/or disabling back problem was considered if a back problem led to either a long-term health problem or limitation of activity (outcome indicator in this study) | Weighted: 7.8 (7.2 to 8.4) |
| HBSC Canada, 1997-1998 [33,34] | National | Multistage clustered  [N = 6,215 (national report, low back pain question); N = 6,567 (respondents in international report)] | NA | 1997-1998 |  | 11-15  52% |  | Frequency of low back pain in the last six months: “In the last six months, how often have you had back pain?” Those who responded “almost every month” or more frequently were considered cases | 49.7 (47.9 to 51.5) |
| Cassidy, 1998 [35] | Subnational (Saskatchewan) | Weighted age-stratified random  (N = 1,131) | 55% | 1995 |  | 20-69  54% |  | The point, six-month, and lifetime prevalence of low back pain was obtained by a direct question, and a mannequin diagram was used to define the anatomic location of low back pain | Point, crude: 28.7 (26.1 to 31.4); Point, age-standardized: 28.4 (25.6 to 31.1); Six months: 71.4 (68.8 to 74.0); Lifetime, age-standardized:  84.1 (81.9 to 86.3) |
| HBSC Canada, 2002 [36,37] | National | Multistage clustered  [N = 4,458 (national report, low back pain question); N = 4,361 (international report)] | NA | 2002 |  | 11-15  54% |  | Frequency of low back pain in the last six months: “In the last six months, how often have you had back pain?” Those who responded “almost every month” or more frequently were considered cases | 41.2 (39.2 to 43.2) |
| Currie, 2004 [38] | National | Multistage stratified random  (N = 118,533) | 80% | 2000-2001 |  | 12-65+  53% |  | Chronic back pain was identified when subjects responded “no” to being free of pain, and endorsing back problems as a long-term health condition in the past 12 months^j^ | 8.9 (8.7 to 9.1) |
| HBSC Canada, 2005 [39,40] | National | Systematic, single-stage cluster  [N = 9,670 (national report); N = 5,787 (international report)] | 74% | 2005-2006 |  | 11-15  53% |  | Frequency of low back pain in the last six months: “In the last six months, how often have you had back pain?” Those who responded “almost every month” or more frequently were considered cases | 46.6 (45.2 to 48.0) |
| HBSC Canada, 2010 [41,42] | National | Two-stage cluster  [N = 26,078 (national report); N = 25,818 (excluding grades 5 and 11); N = 15,710 (international report)] | 77% | 2009-2010 |  | 11-15  50% |  | Frequency of low back pain in the last six months: “In the last six months, how often have you had back pain?” Those who responded “almost every month” or more frequently were considered cases | 44.0 (43.1 to 44.9) |
| Spain |  |  |  |  |  |  |  |  |  |
| Ballina García, 1994 [43] | Subnational (Asturias) | Proportional, multistage, stratified random  (N = 702) | 76% | 1990 |  | 18-65+  54% |  | A “case” was considered when the symptoms had been present in the spine or in the peripheral joints during the year prior to the interview, with a minimum duration of one week | 28.2 (23.9 to 31.0) |
| Carmona, 2001[44,45] | National | Stratified multistage cluster  (N = 2,192) | 73% | 1998-2000 |  | 20-80+  54% |  | Low back pain was defined by self-report. The interviewers were instructed to indicate what was understood by low back and then to ask about (current) pain in that area | 14.8 (12.2 to 17.4) |
| Català, 2002 [46] | National | Multistep, using quotas for sex and age groups for each geographical location stratum  (N = 5,000) | 48% | 1998 |  | 18-65+  52% |  | Individuals with a positive answer to a questionnaire question regarding pain during the previous day were specifically asked about any physical pain during the previous week (open label) | 11.9 (10.6 to 13.2) |
| HBSC Spain, 2002 [47,37] | National | Multistage clustered  [N = 13,552 (sample in national report); N = 5,827 (respondents for Spain in international report)] | NA | 2002 |  | 10-21  50% |  | Frequency of low back pain in the last six months: “In the last six months, how often have you had back pain?” Those who responded “almost every month” or more frequently were considered cases | 42.5 (41.3 to 43.7) |
| WHS Spain, 2002-2003 [48] | National | Multistage cluster  [N = 6,270 (Household);  N = 6,275  (Individual)] | 52%  (Household);  98%  (Individual) | 2002-2003 |  | 15-80+  52% |  | Back pain (including disc problems) in the last 30 days: “Have you experienced back pain (including disc problems) during the last 30 days?” | 35.1 (33.4 to 36.8)^d^ |
| HBSC Spain, 2006 [47,40] | National | Multistage clustered  [N = 21,811 (sample in national report); N = 8,891 (international report)] | NA | 2006 |  | 10-19  52% |  | Frequency of low back pain in the last six months: “In the last six months, how often have you had back pain?” Those who responded “almost every month” or more frequently were considered cases | 38.6 (37.7 to 39.5) |
| Pinto Meza, 2006 [49] | National | Stratified multistage random without replacement  (N = 2,121) | 79% | 2003 |  | 18-65+  61% |  | Self-reported pain was based on the survey questions: 1) “Have you ever experienced chronic thoracic pain or cervical pain?, 2) “Have you experienced chronic thoracic pain or cervical pain in the last 12 months?” | Lifetime: 23.7 (21.0 to 26.4); 12 months: 14.7 (12.3 to 17.1) |
| Demyttenaere, 2007 [50,51] | National | Multistage, clustered area probability household  [N = 42,697 (all countries combined);  N = 2,121 (Spain)] | 70.8% (weighted average across all countries); 79% (Spain) | 2001-2002 |  | 18-60+  51% |  | Self-reported 12-month presence of or treatment for chronic back or neck problems^k^ | Weighted, Spain: 14.7 (12.6 to 16.8) |
| Miró, 2007 [52] | Subnational (Catalonia) | Random  (N = 592) | 95% | 2004 |  | 65-85+  54% |  | Self-reported pain was based on the question: ‘‘In the past 3 months have you had pain that has lasted for one day or longer in any part of your body?’’ | 43.9 (39.9 to 47.9) |
| Pellisé, 2009 [53] | Subnational (Barcelona) | Two-stage cluster (Barcelona),  Unspecified (Fribourg)  [N = 903 (Barcelona);  N = 567 (Fribourg); N = 1,470 (Combined total)] | 85% | 2006 |  | 12–17  47% |  | Pain lasting 1 day or longer during the preceding month in the lumbar area, shown on a pre-shaded manikin | Barcelona: 41.3 (36.8 to 45.8); Fribourg: 37.4 (31.8 to 43.0); Combined total: 39.8 (36.3 to 43.3) |
| HBSC Spain, 2010 [54,42] | National | Multistage stratified clustered  [N = 11,230 (national report);  N = 5,040 (international report)] | NA | 2010 |  | 11-18  51% |  | Frequency of low back pain in the last six months: “In the last six months, how often have you had back pain?” Those who responded “almost every month” or more frequently were considered cases | 38.1 (36.8 to 39.4) |
| Fernández de las Peñas, 2011 [55–57] | National | Probabilistic multistage  (N = 29,478) | 96% | 2006-2007 |  | 16-70+  60% |  | Subjects were classified as low back pain sufferers when they responded “yes” to both of the following questions: 1) “Have you suffered low back pain over the previous 12 months?”, and 2) “Has your physician confirmed the diagnosis?” | 19.9 (19.3 to 20.5) |
| Balagué, 2012 [58] | Subnational (Barcelona) | Two-stage cluster (Barcelona),  Unspecified (Fribourg)  [N = 903 (Barcelona);  N = 567 (Fribourg); N = 1,470 (Combined total)] | 85% | 2008 |  | 12-17  47% |  | Pain lasting 1 day or longer during the preceding month in the lumbar area, shown on a pre-shaded manikin | Barcelona: 41.3 (36.8 to 45.8); Fribourg: 37.4 (31.8 to 43.0); Combined total: 39.8 (36.3 to 43.3) |
| Eurobarometer 66.2 Spain, 2012 [59,60] | National | Multistage random probability  (N = 1,026) | NA | 2006 |  | 15-80+  51% |  | 1) “In the last week, have you had any pain affecting your muscles, joints, neck or back which has affected your ability to carry out the activities of daily living?”, and 2) “have you ever had any pain affecting your muscles, joints, neck or back which has affected your ability to carry out the activities of daily living that lasted for 3 months or more?” Those responding “Yes” and choosing their “low back” as body part were considered cases | One week: 7.9 (6.0 to 9.8); Lifetime chronic: 7.3 (5.4 to 9.2) |
| Jiménez Sánchez, 2012 [61,62] | Subnational (Madrid) | Probabilistic multistage  (N = 12,190) | 38% | 2007 |  | 16-65+  53% |  | Individuals were classified as having chronic low back pain if they responded “yes” to the question “Have you suffered from low back pain over the previous 12 months?” Patients were also asked for medical confirmation of their symptoms | Women: 14.1 (13.2 to 15.0); Men: 7.8 (7.2 to 8.6); Combined: 11.1 (10.5 to 11.7) |
| Rodríguez Oviedo, 2012 [63] | Subnational (Galicia) | Unspecified  (N = 1,403) | 66% | 2005-2006 |  | 12-17  52% |  | Presence of back pain for more than 15 days in the previous year, as ascertained from parents or legal tutors | 25.9 (22.7 to 29.1) |
| Vargas Prada, 2013 [64] | Subnational (Barcelona) | Unspecified  (N = 1,105) | 97% | 2007-2010 |  | 20-59  88% |  | Low back pain was ascertained through a question which asked whether, during the past 12 months, pain had been present for a day or longer in an anatomical area between the twelfth ribs and the gluteal folds, which was depicted in a diagram^l^ | 12 months: 63.6 (59.6 to 67.6); 1 month: 41.6 (37.5 to 45.7) |
| Mesas, 2014 [57,65] | National | Probabilistic multistage  (N = 8,283) | 96% | 2009-2010 |  | 18-70  43% |  | Chronic back pain in the past 12 months: “Have you suffered chronic back pain in the past 12 months?” | 14.1 (13.4 to 14.8) |
| Koyanagi, 2015 [66,67] | National | Stratified multistage clustered  (N = 3,625) | 70% (Wave 1, baseline) | 2011-2012 |  | 50-80+  (51%) |  | Information on back pain was obtained by asking “Have you experienced back pain during the last 30 days?” | Age-sex adjusted: 43.5 (40.3 to 46.7);  Crude: 45.1 (42.2 to 48.0) |
| Switzerland |  |  |  |  |  |  |  |  |  |
| Balagué, 1994 [4] | Subnational (Fribourg) | Unspecified  (N = 1,716) | 98% | 1989 |  | 8-16  51% |  | Children who reported having had at least one episode of back pain localized to the lower back were classified as low back pain | 1 week: 12.0 (10.4 to 13.6); Lifetime: 17.7 (15.8 to 19.5) |
| HBSC Switzerland, 1998 [34] | National | Cluster  [N = 5,520 (international report)] | NA | 1997-1998 |  | 11-15  50% |  | Frequency of low back pain in the last six months: “In the last six months, how often have you had back pain?” Those who responded “once a week” or more frequently were considered cases | 11 year old females: 12.0%; 11 year old males: 8.0%; 13 year old females: 19.0%; 13 year old males: 13.0%; 15 year old females: 23.0%; 15 year old males: 19.0% |
| SHP, 1999-2000 [68,69] (Wave 1) | National | Stratified random  [N = 5,074 (Household); N = 7,799 (Individual)] | Wave 1: 64%  (Household);  85% (Individual, conditional upon household participation) | 1999 |  | 13-90+  51% |  | “Back problems” in the last 12 months | 34.2 (33.1 to 35.3) |
| Santos-Eggimann, 2000 [70] | Subnational (Vaud, Fribourg, and Ticino) | Probabilistic, two-stage  (N = 1,718 in Vaud-Fribourg; N = 1,509 in Ticino; N = 3,227 combined) | 52% (Vaud-Firbourg);  76% (Ticino) | 1992-1993 |  | 25-74  NA |  | According to the definition of low back pain in the Nordic questionnaire, subjects were asked to report any ache, pain, or discomfort located in the lower back (indicated by the shaded area on a diagram), with or without radiation to one or both legs (sciatica) during the preceding 12 months^m^ | Lasting >7 and >30 cumulated days (combined range): 9.5-38.5 (5.4 to 48.0) |
| SHP, 2000-2001 [68,69] (Wave 1) | National | Stratified random  [N = 4,425 (Household); N = 7,073 (Individual)] | Wave 1: 64%  (Household);  85%  (Individual, conditional upon household participation) | 2000 |  | 13-90+  51% |  | “Back problems” in the last 12 months | 38.7 (37.6 to 39.8) |
| SHP, 2001-2002 [68,69] (Wave 1) | National | Stratified random  [N = 4139 (Household);  N = 6601  (Individual)] | Wave 1: 64%  (Household);  85%  (Individual, conditional upon household participation) | 2001 |  | 13-90+  51% |  | “Back problems” in the last 12 months | 38.4 (37.2 to 39.6) |
| HBSC Switzerland, 2002 [71,37] | National | Cluster  [N = 9275 (unweighted, national report);  N = 4,679 (international report)] | NA | 2002 |  | 11-15  51% |  | Frequency of low back pain in the last six months: “In the last six months, how often have you had back pain?” Those who responded “almost every month” or more frequently were considered cases | 38.6 (37.2 to 40.0) |
| SHP, 2002-2003 [68,69] (Wave 1) | National | Stratified random  [N= 3,582  (Household);  N = 5,700  (Individual)] | Wave 1: 64%  (Household);  85%  (Individual, conditional upon household participation) | 2002 |  | 13-90+  51% |  | “Back problems” in the last 12 months | 37.8 (36.5 to 39.1) |
| SHP, 2003-2004 [68,69] (Wave 1) | National | Stratified random  [N = 3,227 (Household); N = 5,220 (Individual)] | Wave 1: 64%  (Household); 85%  (Individual, conditional upon household participation) | 2003-2004 |  | 13-90+  51% |  | “Back problems” in the last 12 months | 35.8 (34.5 to 37.1) |
| SHP, 2004-2005 [68,69] (Waves 1 and 2) | National | Stratified random  [N = 5,374 (Household);  N = 8,065  (Individual)] | Wave 1: 64%  (Household);  85%  (Individual, conditional upon household participation);  Wave 2: 65%  (Household);  76%  (Individual, conditional upon household participation) | 2005-2006 |  | 13-90+  51% |  | “Back problems” in the last 4 weeks^n^ | 44.8 (43.7 to 45.9) |
| HBSC Switzerland, 2006 [72,40] | National | Cluster  [N = 9,507 (unweighted, national report); N = 4,579 (international report)] | 86% | 2006 |  | 11-15  51% |  | Frequency of low back pain in the last six months: “In the last six months, how often have you had back pain?” Those who responded “almost every month” or more frequently were considered cases | 42.4 (41.0 to 43.8) |
| SHP, 2006-2007 [68,69] (Waves 1 and 2) | National | Stratified random  [N = 4,220 (Household); N = 6,657 (Individual)] | Wave 1: 64%  (Household); 85%  (Individual, conditional upon household participation);  Wave 2: 65%  (Household); 76%  (Individual, conditional upon household participation) | 2006 |  | 14-90+  51% |  | “Back problems” in the last 4 weeks^n^ | 44.7 (43.5 to 45.9) |
| SHP, 2007-2008 [68,69] (Waves 1 and 2) | National | Stratified random  [N = 4,310 (Household); N = 6,979 (Individual)] | Wave 1: 64%  (Household);  85%  (Individual, conditional upon household participation);  Wave 2: 65%  (Household);  76%  (Individual, conditional upon household participation) | 2007 |  | 14-90+  51% |  | “Back problems” in the last 4 weeks^n^ | 45.3 (44.1 to 46.5) |
| SHP, 2008-2009 [68,69] (Waves 1 and 2) | National | Stratified random  [N = 4,263 (Household); N = 6,903 (Individual)] | Wave 1: 64%  (Household);  85%  (Individual, conditional upon household participation);  Wave 2: 65%  (Household);  76%  (Individual, conditional upon household participation) | 2008 |  | 14-90+  52% |  | “Back problems” in the last 4 weeks^n^ | 44.8 (43.6 to 46.0) |
| Pellisé, 2009 [53] | Subnational (Fribourg) | Two-stage cluster (Barcelona),  Unspecified (Fribourg)  [N = 903 (Barcelona);  N = 567 (Fribourg); N = 1,470 (Combined total)] | 85% | 2006 |  | 12–17  47% |  | Pain lasting 1 day or longer during the preceding month in the lumbar area, shown on a pre-shaded manikin | Barcelona: 41.3 (36.8 to 45.8); Fribourg: 37.4 (31.8 to 43.0); Combined total: 39.8 (36.3 to 43.3) |
| HBSC Switzerland, 2010 [73,42] | National | Cluster  [N = 9,886 (unweighted, national report); N = 6,611 (international report)] | 88% | 2010 |  | 11-15  50% |  | Frequency of low back pain in the last six months: “In the last six months, how often have you had back pain?” Those who responded “almost every month” or more frequently were considered cases | 41.8 (40.4 to 43.1) |
| Erne, 2011 [74] | Subnational (Aargau) | Unspecified  (N = 189) | 47% (School);  88% (Student) | 2008 |  | 10-13  55% |  | Body pain for a day or longer in the last 4 weeks: “Did you feel pain for a day or even longer in the last 4 weeks? If yes, please paint on that body figure those areas where you felt pain for a day or even longer” | 13.8 (6.8 to 20.8) |
| Kolb, 2011 [75]^,d^ | National | Random stratified  (N = 3,881) | 64% (Household);  85% (Individual, conditional upon household participation) | 1999-2003 |  | 13-90+  58% |  | The back pain question asked about “bad back” or “lower back problems” in the last 12 months with a frequency of at least once a month: “In the last 12 months, have you suffered at least once a month from bad back or lower back problems?” | Year 1999: 33.2 (31.7 to 34.7); Year 2000: 38.5 (37.0 to 40.0); Year 2001: 37.4 (35.9 to 38.9); Year 2002: 38.0 (36.5 to 39.5); Year 2003: 37.0 (35.5 to 38.5) |
| Balagué, 2012 [58] | Subnational (Fribourg) | Two-stage cluster (Barcelona),  Unspecified (Fribourg)  [N = 903 (Barcelona);  N = 567 (Fribourg); N = 1,470 (Combined total)] | 85% | 2008 |  | 12-17  47% |  | Pain lasting 1 day or longer during the preceding month in the lumbar area, shown on a pre-shaded manikin | Barcelona: 41.3 (36.8 to 45.8); Fribourg: 37.4 (31.8 to 43.0); Combined total: 39.8 (36.3 to 43.3) |

Abbreviations: *CATI*: Computer-assisted telephone interviewing; *DC*: Drop and collect; *HBSC*, Health Behaviour in School-aged Children; *NA*, Not available; *NHS*, National Health Survey; *SDAC,* Survey of Disability, Ageing and Carers; *SHP*, Swiss Household Panel; *WHS*, World Health Survey

^a^ NHS: Proportions were age standardized to the 2001 Australian population to account for differences in the age structure of the population over time.

^b^ SDAC: Household component included 14,019 private dwellings and 303 non-private dwelling units in 2003, and 27,600 private dwellings and 200 non-private dwelling units in 2009.

^c^ SDAC: Cared-accommodation component included 564 cared-accommodation establishments in 2003, and 1100 cared accommodation establishments in 2009.

^d^ WHS: Based on question Q6007 in the questionnaire. Q6007 attempts to identify people who have back pain that could be related to osteoarthritis or other problems such as spinal trauma and disc displacement in the spine. The WHO discourages the interpretation of this estimate as a summary statistic of the population of interest.

^e^ O’Sullivan: Low back pain in the sample was characterized by the formation of three mutually exclusive low back pain prevalence groups according to answers to three questions: 1) no current low back pain, 2) current non-chronic low back pain 3) current chronic low back pain.

^f^ Mendoza-Sassi: For each health problem, subjects were asked if any measures were being taken. If so, they were asked if they were self-medicating or receiving medical care.

^g^ Ferreira: In case of a positive response, the subject had to indicate the site in a coloured human picture that differentiated the cervical, thoracic, and lumbosacral regions.

^h^ Meziat Filho: Subjects were classified as “chronic low back pain” cases when they answered “yes” in the first and second questions. When the answer was “yes” only in the first, they were classified as “acute low back pain” cases.

^i^ Noll: 158 chose the alternative “I don’t know” and were excluded from the analysis by the study authors (i.e., they were not included in the denominator).

^j^ Currie: The definition of a long-term health condition included two criteria: (1) the condition was diagnosed by a health professional and (2) it had lasted at least 6 months.

^k^ Demyttenaere: Adapted from the US Health Interview Survey. If affirmative, respondents were asked if they had had back or neck problems in the last 12 months.

^l^ Vargas Prada: Those who answered “yes” were asked whether the pain had been present for more than four weeks in total, whether it had been present in the past month, and whether during the past month it had made it difficult or impossible to cut toe nails, get dressed or do normal jobs around the house .

^m^ Santos-Eggimann: Participants reported their experience of low back pain, if any, and specified the cumulative duration of low back pain during the preceding 12 months in predetermined categories abstracted from the Nordic questionnaire: 1 to 7 days, 8 to 30 days, more than 30 days but not every day, and every day.

^n^ SHP: Categorical variable (categories: “Not at all”, “Somewhat”, and “Very much”). Only those who answered “Somewhat” and “Very much” were considered cases.

**sTable 3.** Detailed evidence table of primary data input studies informing GBD 2019 modelled epidemiologic estimates of neck pain in Australia, Brazil, Canada, Spain, and Switzerland.

| Study, year | Sampling frame | Sampling design (Sample size, N) | Response (%) | Years covered | Ages (y)  Women (%) | Case definition | Prevalence estimate % (95% CI) |
| --- | --- | --- | --- | --- | --- | --- | --- |
| Australia | | | | | | | |
| No primary data input studies available | | | | | | | |
| Brazil | | | | | | | |
| Genebra, 2017 [76] | Subnational (Bauru) | Two-stage cluster  (N = 600) | 94% | 2012 | 20-60+  50% | Pain, ache, or discomfort in the area between the occiput and the third thoracic vertebra and between the medial borders of the scapulae in the past year^a^ | 20.3 (17.3 to 23.7) |
| Canada | | | | | | | |
| No primary data input studies available | | | | | | | |
| Spain | | | | | | | |
| Jiménez Sánchez, 2012 [61,62] | Subnational (Madrid) | Probabilistic multistage  (N = 12,190) | 38% | 2007 | 16-65+  53% | Individuals were classified as having chronic neck pain if they responded “yes” to the question “Have you suffered from neck pain over the previous 12 months?”^b^ | Women: 8.4 (7.7 to 9.1); Men: 3.2 (2.8 to 3.7); Combined: 6.0 (5.6 to 6.4)^c^ |
| Switzerland | | | | | | | |
| No primary data input studies available | | | | | | | |
| ^a^ Neck pain was measured using the Nordic questionnaire, validated and adapted to the Brazilian culture.[77] In the interview, individuals were asked the following question: “Did you have any pain or discomfort in the neck in the past year?” In addition to the verbal questionnaire, an image of the spinal regions in different colors was presented, so the interviewees could better specify the neck region that was painful. ^b^ Study participants were asked for medical confirmation of their symptoms. ^c^ Combined total prevalence estimate derived from sex-specific chronic neck pain prevalence estimates | | | | | | | |

**sTable 4.** Detailed evidence table of primary data input studies informing GBD 2019 modelled epidemiologic estimates of knee osteoarthritis in Australia, Brazil, Canada, Spain, and Switzerland.

| Study, year | Sampling frame | Sampling design (Sample size, N) | Response (%) | Years covered | Ages (y) Women (%) | Case definition | Prevalence estimate % (95% CI) |
| --- | --- | --- | --- | --- | --- | --- | --- |
| Australia | | | | | | | |
| No primary data input studies available | | | | | | | |
| Brazil | | | | | | | |
| No primary data input studies available | | | | | | | |
| Canada | | | | | | | |
| Plotnikoff, 2015 [78,79] | Subnational (Alberta) | Random digit dial  [N = 4,733 (Phase 1); N = 1,808 (Phase 2)] | 38%^a^ | 2009-2010 | 15-65+  53% | Self -reported: “Yes” or “no” to the question, “Do you have knee osteoarthritis?”^b^  Clinic interviews: “Yes” or “no” to a validated questionnaire^c^ relating to knee OA | Self-reported: 10.5 (9.3 to 11.7); Robust^d^ men, weighted: 4.4 (3.2 to 5.6); Robust^d^ women, weighted: 6.7 (5.3 to 8.1) |
| Spain | | | | | | | |
| Carmona, 2001 [44,45] | National | Stratified multistage cluster  (N = 2,192) | 73% | 1998 | 20-80+  54% | ACR definition of knee osteoarthritis^e^ by clinical criteria[80] | 10.2 (8.5 to 11.9) |
| Fernández López, 2008 [81] | National | Multistage cluster  (N = 2,192) | 73% | 2000 | 20-80+  54% | ACR definition of knee osteoarthritis^e^ by clinical criteria[80] | 10.2 (7.9 to 12.5) |
| Switzerland |  |  |  |  |  |  |  |
| No primary data input studies available | | | | | | | |
| Abbreviations: *ACR*, American College of Rheumatology. ^a^ Proportion of phase 1 participants agreeing to take part in phase 2. ^b^ Response options “Do not know” or “Refuse to answer” were also provided during the phone interviews. ^c^ Participants were asked: During the past 4 weeks, have you had knee pain on most days?; During the past 4 weeks, have you had knee pain while climbing down stairs or walking down slopes?; During the past 4 weeks, have you had swelling in one or both knees?; Do you have knee osteoarthritis?; If you do, was the diagnosis made by a rheumatologist or a general practitioner? ^d^ Robust values represent participants responding “yes” to having OA through both the phone survey and clinic survey, and indicating their OA was diagnosed by a general practice physician or rheumatologist. ^e^ A person was classified as having knee pain suggesting osteoarthritis if they answered affirmatively to “Have you had pain on either knee for most of the time in the previous month?” and also presented four or more of the following criteria: 1) age over 50, 2) morning stiffness shorter than 30 minutes, 3) knee crepitus on active joint motion, 4) pain when making pressure at bony margins of the joint, 5) bony joint enlargement, and 6) absence of clear signs of inflammation | | | | | | | |

**sFigure 4.** Traffic-light plot representing the risk of bias assessment of primary data input studies informing GBD 2019 modelled prevalence estimates of low back pain, neck pain, and knee osteoarthritis in Australia, Brazil, Canada, Spain, and Switzerland (created with {robvis} in R [82]).


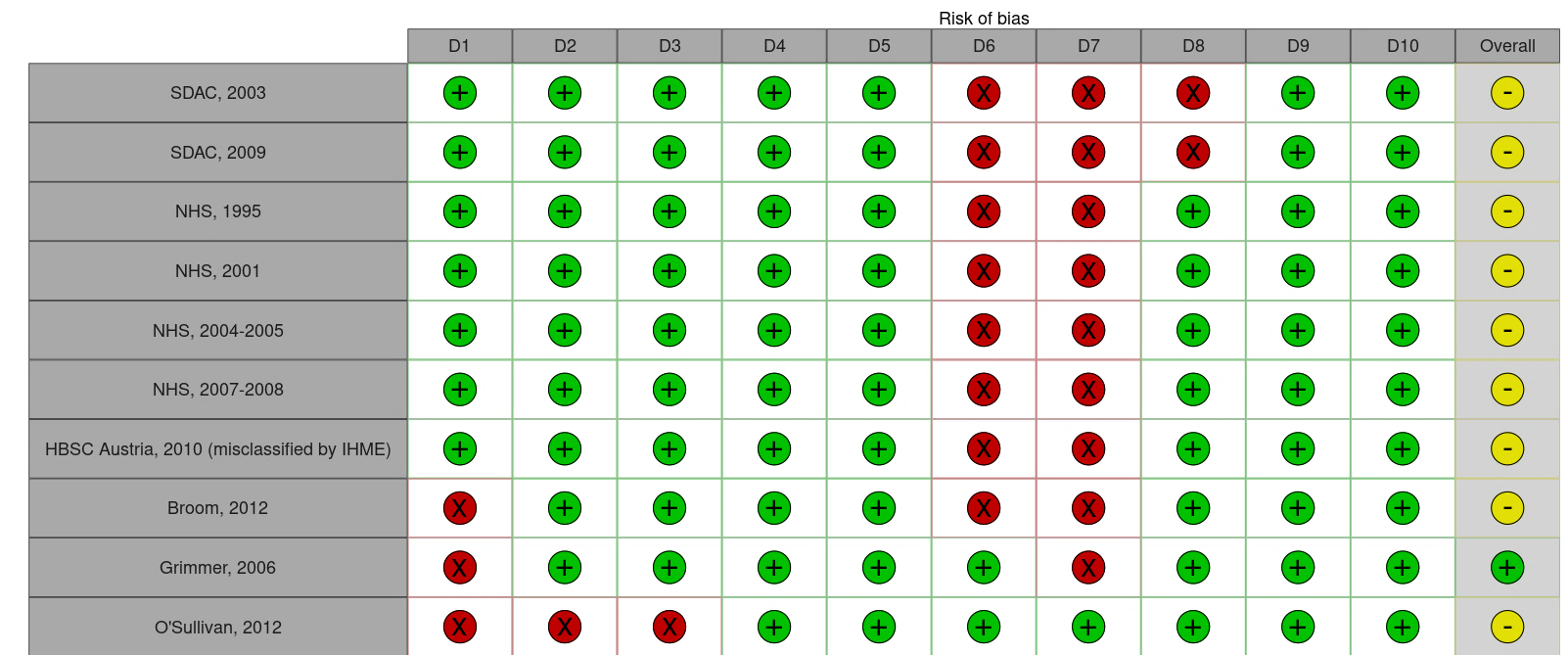


**
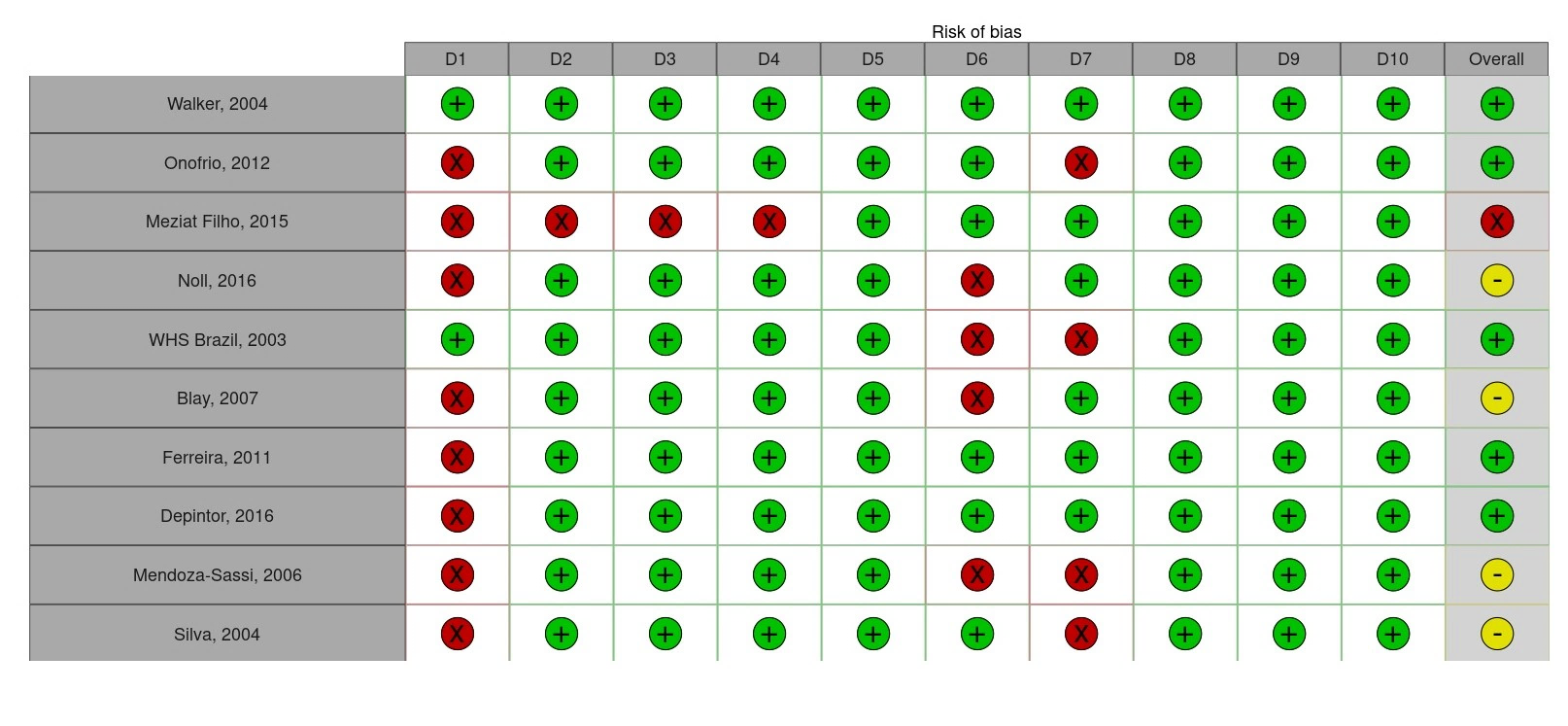
**

**
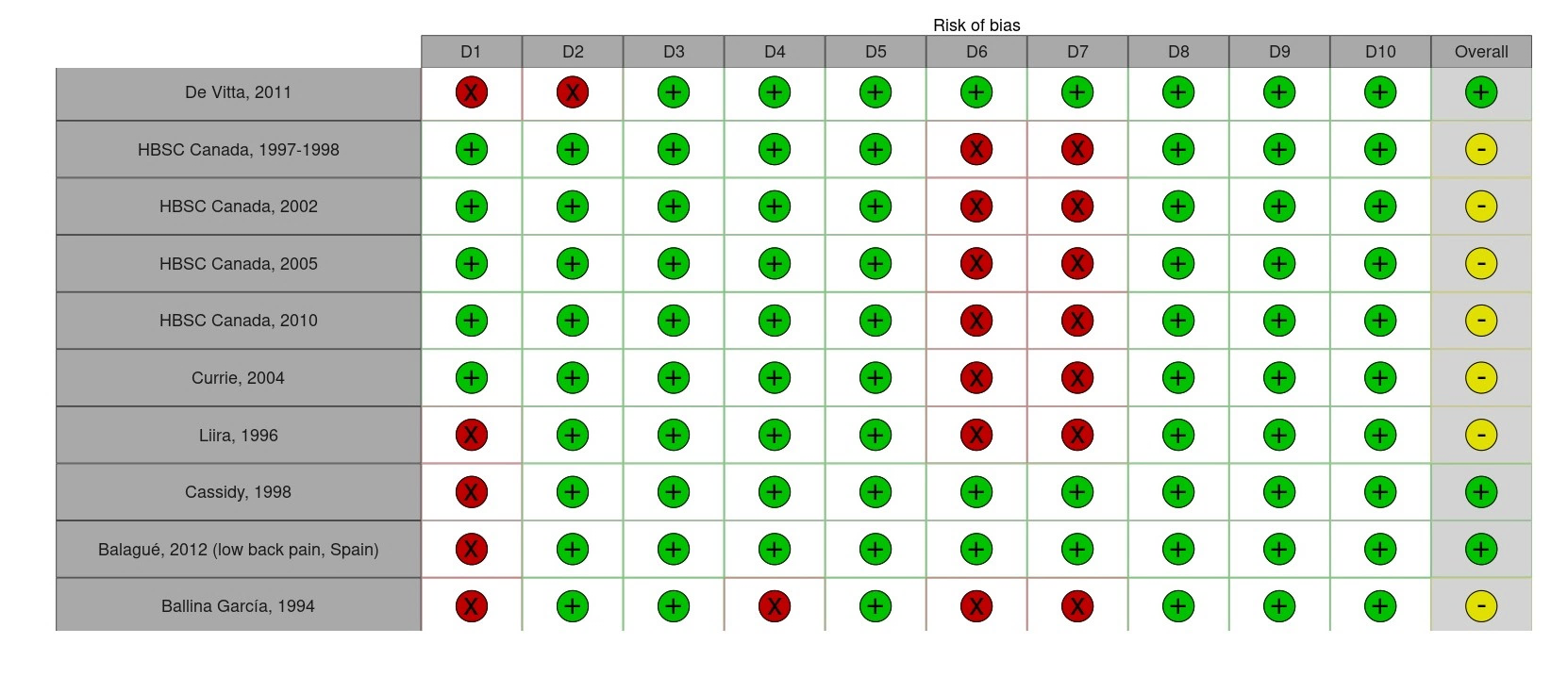
**

**
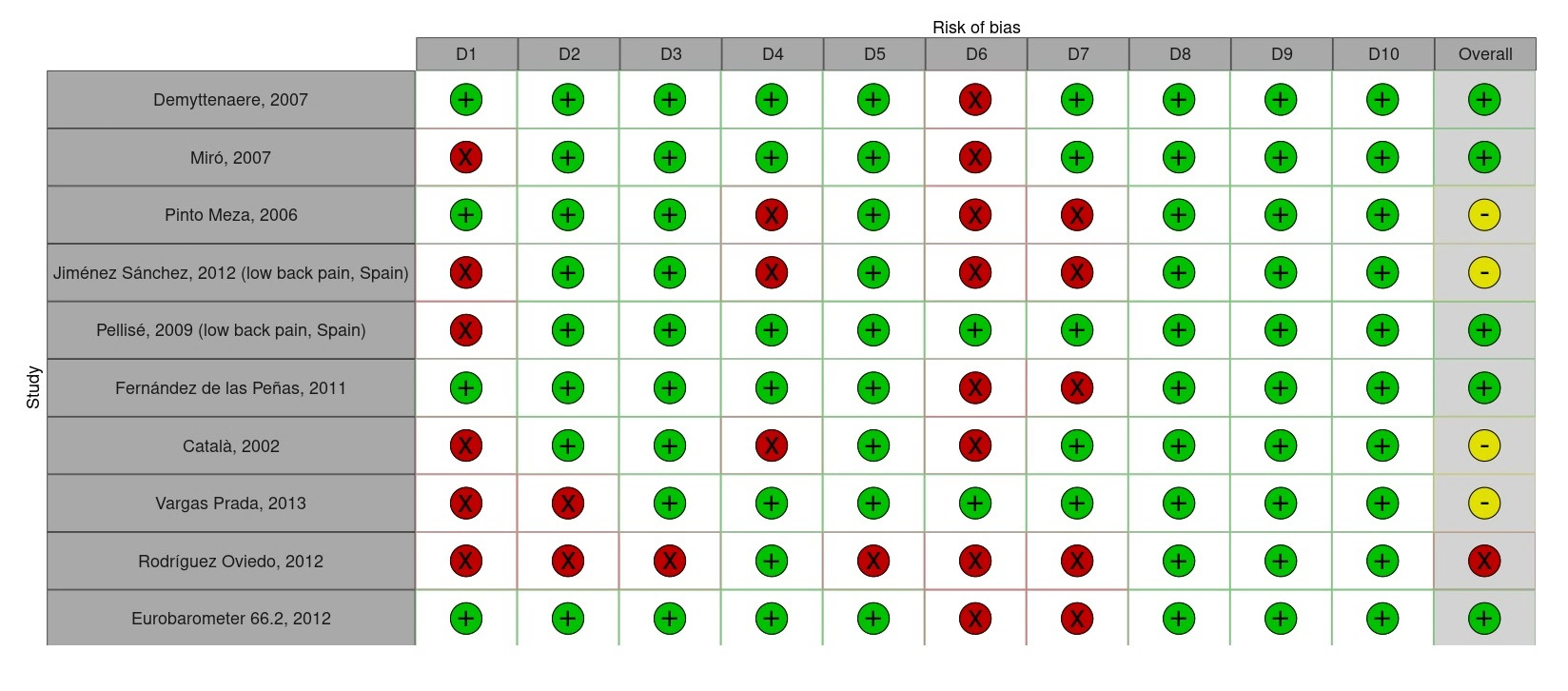
**

**
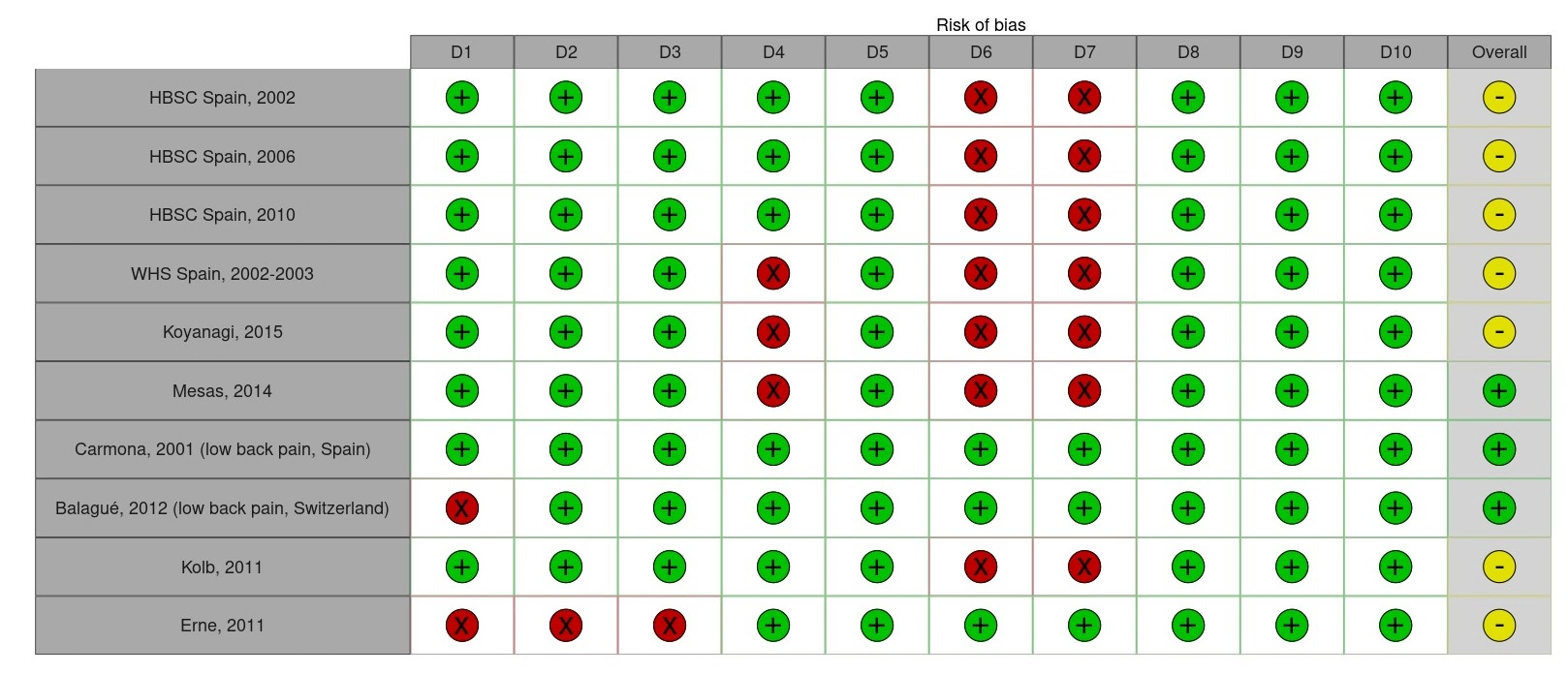
**

**
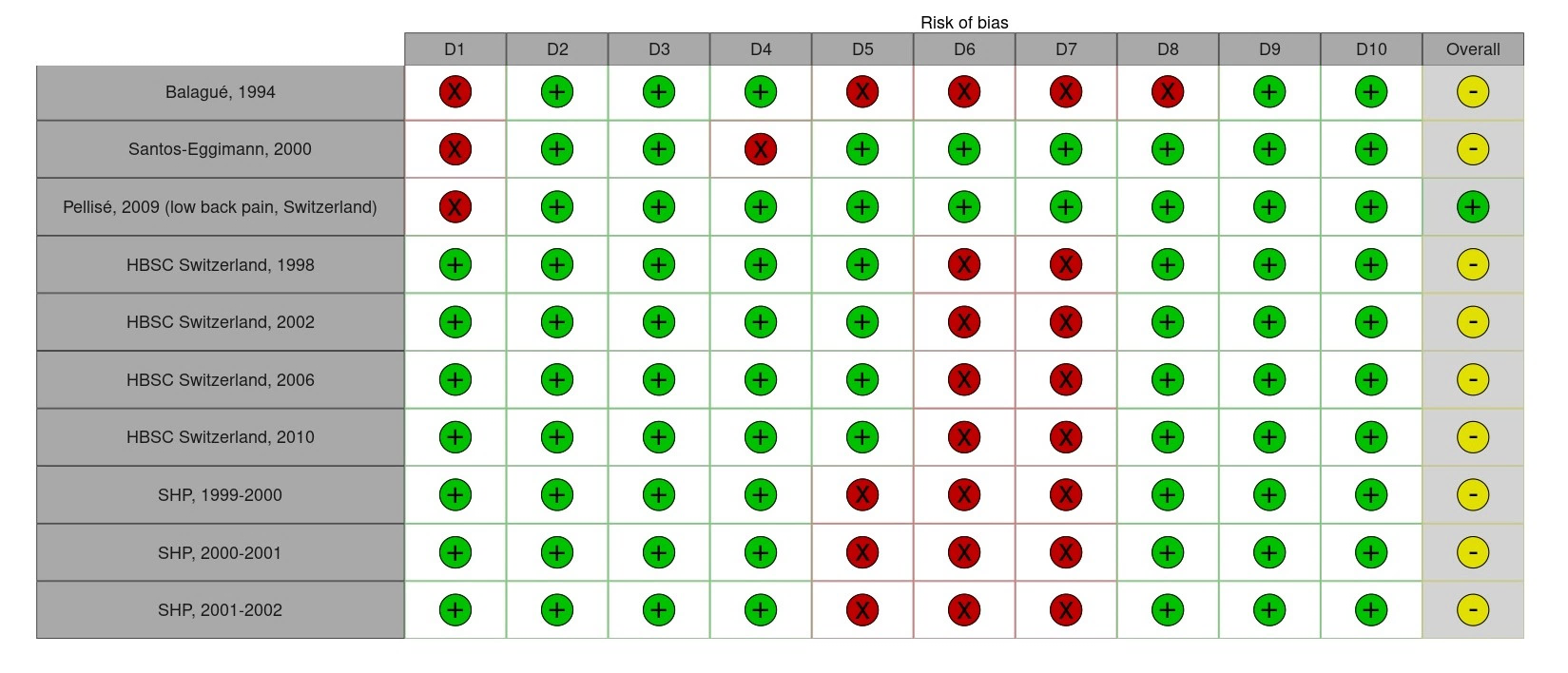
**

**
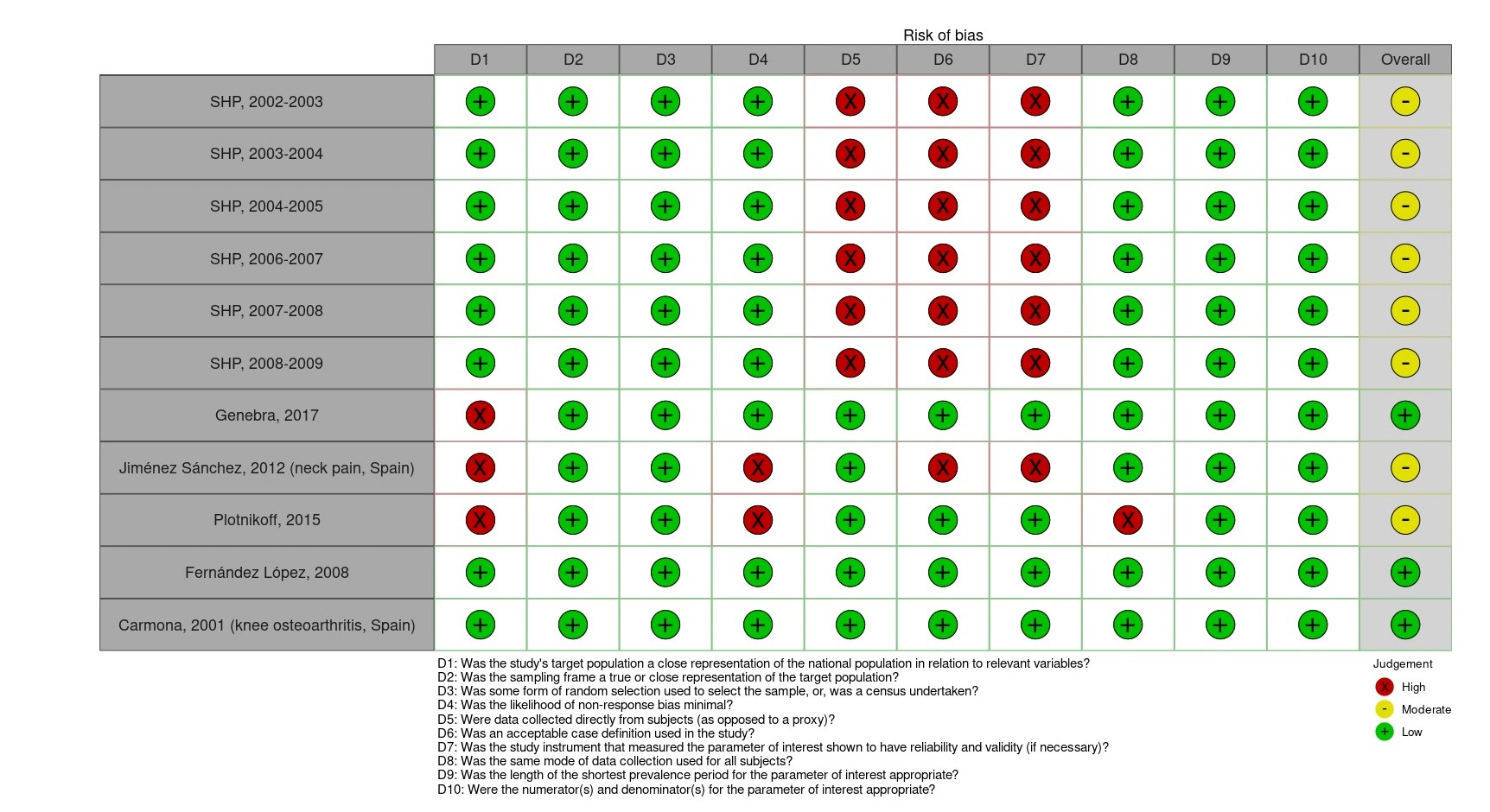
**

**sFigure 5.** Summary plot representing the risk of bias assessment of primary data input studies informing GBD 2019 modelled prevalence estimates of low back pain, neck pain, and knee osteoarthritis in Australia, Brazil, Canada, Spain, and Switzerland (created with {robvis} in R [82]).

[
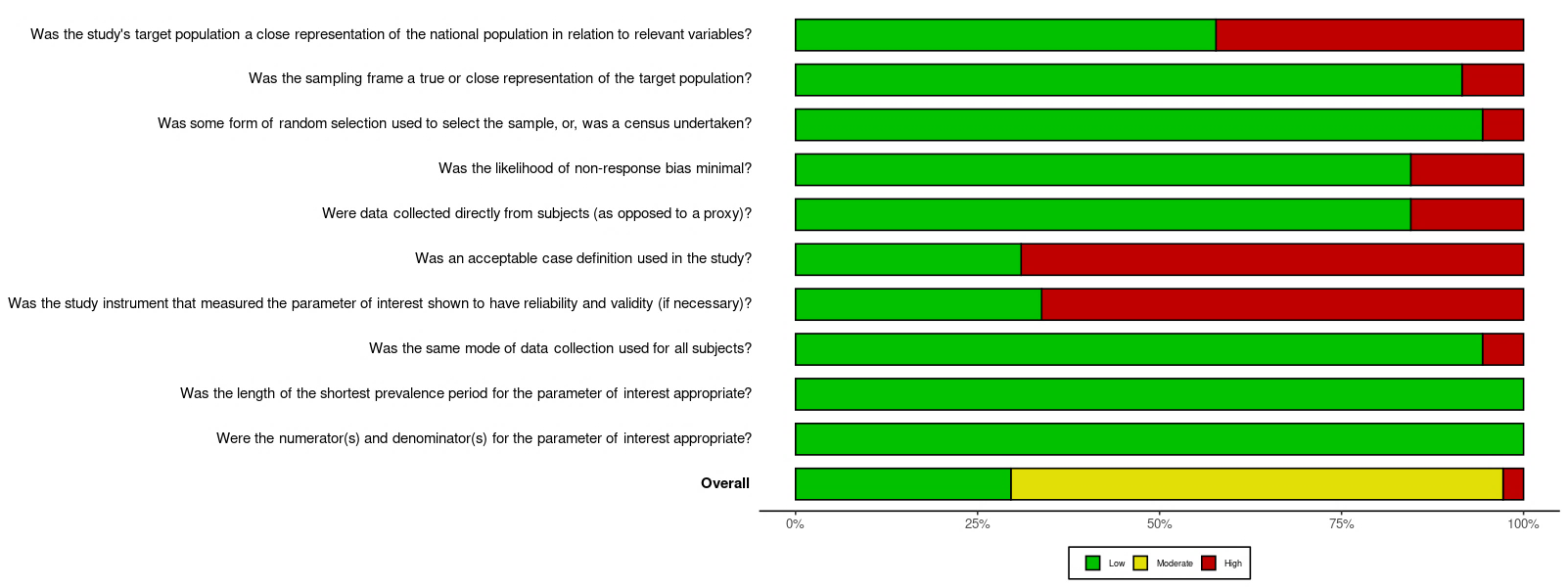
](file:///C:\Users\14084\Desktop\Master's%20thesis%20UAM\R_shiny_robvis\summary_plot_07_24_2022.png)

| sTable 5. GBD 2019 modelled prevalence^a^ estimates for low back pain in Australia, Brazil, Canada, Spain, and Switzerland from 1990 to 2019. | | | | | | | | | |
| --- | --- | --- | --- | --- | --- | --- | --- | --- | --- |
| Year | **Prevalence estimate % (95% UI)** | **Year** | **Prevalence estimate % (95% UI)** | **Year** | **Prevalence estimate % (95% UI)** | **Year** | **Prevalence estimate % (95% UI)** | **Year** | **Prevalence estimate % (95% UI)** |
| Australia | | | | |  | | | | |
| 1990 | 11.6 (10.4 to 12.9) | 1996 | 11.8 (10.7 to 13.1) | 2002 | 12.1 (10.8 to 13.4) | 2008 | 12.4 (11.2 to 13.8) | 2014 | 11.7 (10.6 to 13.1) |
| 1991 | 11.7 (10.5 to 12.9) | 1997 | 11.8 (10.6 to 13.1) | 2003 | 12.2 (11.0 to 13.5) | 2009 | 12.4 (11.2 to 13.8) | 2015 | 11.6 (10.5 to 13.0) |
| 1992 | 11.7 (10.5 to 12.9) | 1998 | 11.8 (10.6 to 13.1) | 2004 | 12.3 (11.1 to 13.6) | 2010 | 12.4 (11.2 to 13.9) | 2016 | 11.6 (10.5 to 13.0) |
| 1993 | 11.7 (10.6 to 12.9) | 1999 | 11.8 (10.6 to 13.1) | 2005 | 12.3 (11.1 to 13.7) | 2011 | 12.3 (11.2 to 13.8) | 2017 | 11.6 (10.4 to 13.1) |
| 1994 | 11.7 (10.6 to 13.0) | 2000 | 11.9 (10.6 to 13.2) | 2006 | 12.4 (11.2 to 13.7) | 2012 | 12.1 (11.0 to 13.5) | 2018 | 11.7 (10.4 to 13.2) |
| 1995 | 11.8 (10.7 to 13.0) | 2001 | 11.9 (10.7 to 13.3) | 2007 | 12.4 (11.2 to 13.8) | 2013 | 11.9 (10.7 to 13.3) | 2019 | 11.7 (10.2 to 13.3) |
| Brazil | | | | | | | | | |
| 1990 | 6.7 (5.8 to 7.6) | 1996 | 7.2 (6.3 to 8.2) | 2002 | 7.6 (6.6 to 7.6) | 2008 | 8.0 (7.0 to 9.0) | 2014 | 8.3 (7.2 to 9.3) |
| 1991 | 6.8 (5.9 to 7.7) | 1997 | 7.3 (6.3 to 8.3) | 2003 | 7.6 (6.7 to 8.7) | 2009 | 8.0 (7.0 to 9.0) | 2015 | 8.3 (7.2 to 9.4) |
| 1992 | 6.9 (6.0 to 7.8) | 1998 | 7.3 (6.4 to 8.3) | 2004 | 7.7 (6.7 to 8.7) | 2010 | 8.1 (7.0 to 9.1) | 2016 | 8.3 (7.3 to 9.4) |
| 1993 | 7.0 (6.1 to 7.9) | 1999 | 7.4 (6.5 to 8.4) | 2005 | 7.8 (6.8 to 8.8) | 2011 | 8.1 (7.1 to 9.2) | 2017 | 8.4 (7.3 to 9.5) |
| 1994 | 7.1 (6.2 to 8.0) | 2000 | 7.5 (6.5 to 8.5) | 2006 | 7.8 (6.8 to 8.9) | 2012 | 8.2 (7.1 to 9.2) | 2018 | 8.5 (7.5 to 9.6) |
| 1995 | 7.2 (6.2 to 8.1) | 2001 | 7.5 (6.6 to 8.5) | 2007 | 7.9 (6.9 to 8.9) | 2013 | 8.2 (7.2 to 9.3) | 2019 | 8.7 (7.6 to 9.9) |
| Canada | | | | | | | | | |
| 1990 | 10.1 (9.8 to 10.5) | 1996 | 10.2 (9.9 to 10.6) | 2002 | 10.5 (10.2 to 10.9) | 2008 | 10.8 (10.5 to 11.1) | 2014 | 10.8 (10.5 to 11.2) |
| 1991 | 10.2 (9.9 to 10.5) | 1997 | 10.3 (10.0 to 10.6) | 2003 | 10.6 (10.3 to 10.9) | 2009 | 10.8 (10.5 to 11.1) | 2015 | 10.9 (10.6 to 11.2) |
| 1992 | 10.2 (9.9 to 10.5) | 1998 | 10.3 (10.0 to 10.6) | 2004 | 10.6 (10.4 to 11.0) | 2010 | 10.8 (10.5 to 11.1) | 2016 | 11.6 (10.9 to 12.4) |
| 1993 | 10.2 (9.9 to 10.5) | 1999 | 10.3 (10.0 to 10.7) | 2005 | 10.7 (10.4 to 11.1) | 2011 | 10.8 (10.5 to 11.1) | 2017 | 12.3 (10.8 to 14.1) |
| 1994 | 10.2 (9.9 to 10.5) | 2000 | 10.4 (10.1 to 10.7) | 2006 | 10.7 (10.5 to 11.1) | 2012 | 10.8 (10.5 to 11.1) | 2018 | 12.4 (10.9 to 14.2) |
| 1995 | 10.2 (9.9 to 10.6) | 2001 | 10.5 (10.2 to 10.8) | 2007 | 10.8 (10.5 to 11.1) | 2013 | 10.8 (10.5 to 11.2) | 2019 | 12.4 (10.8 to 14.2) |
| Spain | | | | | | | | | |
| 1990 | 11.1 (10.5 to 11.8) | 1996 | 10.2 (9.5 to 11.0) | 2002 | 10.5 (9.7 to 11.3) | 2008 | 10.5 (9.7 to 11.2) | 2014 | 10.3 (9.4 to 11.4) |
| 1991 | 10.8 (10.1 to 11.5) | 1997 | 10.3 (9.5 to 11.1) | 2003 | 10.5 (9.8 to 11.3) | 2009 | 10.5 (9.7 to 11.3) | 2015 | 10.3 (9.3 to 11.5) |
| 1992 | 10.5 (9.8 to 11.2) | 1998 | 10.3 (9.6 to 11.2) | 2004 | 10.5 (9.7 to 11.3) | 2010 | 10.4 (9.7 to 11.3) | 2016 | 10.2 (9.1 to 11.4) |
| 1993 | 10.3 (9.6 to 11.1) | 1999 | 10.4 (9.7 to 11.3) | 2005 | 10.5 (9.7 to 11.3 | 2011 | 10.4 (9.6 to 11.3) | 2017 | 10.1 (9.0 to 11.5) |
| 1994 | 10.1 (9.4 to 10.9) | 2000 | 10.5 (9.7 to 11.3) | 2006 | 10.5 (9.7 to 11.3) | 2012 | 10.4 (9.5 to 11.3) | 2018 | 10.3 (9.2 to 11.7) |
| 1995 | 10.1 (9.4 to 11.1) | 2001 | 10.5 (9.7 to 11.3) | 2007 | 10.5 (9.7 to 11.3) | 2013 | 10.3 (9.4 to 11.4) | 2019 | 10.6 (9.4 to 12.2) |
| Switzerland | | | | | | | | | |
| 1990 | 18.1 (17.3 to 19.0) | 1996 | 18.0 (17.3 to 18.9) | 2002 | 18.4 (17.6 to 19.2) | 2008 | 18.9 (18.1 to 19.7) | 2014 | 19.2 (18.3 to 20.2) |
| 1991 | 18.1 (17.3 to 18.9) | 1997 | 18.1 (17.3 to 18.9) | 2003 | 18.5 (17.7 to 19.3) | 2009 | 19.0 (18.2 to 19.8) | 2015 | 19.2 (18.2 to 20.3) |
| 1992 | 18.0 (17.3 to 18.9) | 1998 | 18.2 (17.4 to 19.0) | 2004 | 18.6 (17.8 to 19.4) | 2010 | 19.0 (18.2 to 19.9) | 2016 | 19.0 (18.0 to 20.2) |
| 1993 | 18.0 (17.2 to 18.8) | 1999 | 18.2 (17.4 to 19.1) | 2005 | 18.7 (17.9 to 19.5) | 2011 | 19.1 (18.2 to 19.9) | 2017 | 18.6 (17.4 to 19.9) |
| 1994 | 18.0 (17.2 to 18.8) | 2000 | 18.3 (17.5 to 19.1) | 2006 | 18.7 (17.9 to 19.6) | 2012 | 19.1 (18.3 to 20.0) | 2018 | 17.3 (16.0 to 18.9) |
| 1995 | 18.0 (17.3 to 18.9) | 2001 | 18.4 (17.5 to 19.2) | 2007 | 18.8 (18.0 to 19.7) | 2013 | 19.2 (18.3 to 20.1) | 2019 | 15.0 (13.1 to 17.2) |
| Abbreviations: *UI*, Uncertainty Interval  ^a^ Prevalence: The proportion of people in a population who are a case of a disease, injury or sequela. All results in GBD refer to point prevalence  Global Burden of Disease Collaborative Network. Global Burden of Disease Study 2019 (GBD 2019) Results. Seattle, United States: Institute for Health Metrics and Evaluation (IHME), 2020. Available from <https://vizhub.healthdata.org/gbd-results/> | | | | | | | | | |

| sTable 6. GBD 2019 modelled prevalence^a^ estimates for neck pain in Australia, Brazil, Canada, Spain, and Switzerland from 1990 to 2019. | | | | | | | | | |
| --- | --- | --- | --- | --- | --- | --- | --- | --- | --- |
| Year | **Prevalence estimate % (95% UI)** | **Year** | **Prevalence estimate % (95% UI)** | **Year** | **Prevalence estimate % (95% UI)** | **Year** | **Prevalence estimate % (95% UI)** | **Year** | **Prevalence estimate % (95% UI)** |
| Australia | | | | |  | | | | |
| 1990 | 1.3 (1.0 to 1.6) | 1996 | 1.4 (1.1 to 1.7) | 2002 | 1.4 (1.1 to 1.8) | 2008 | 1.4 (1.1 to 1.8) | 2014 | 1.5 (1.2 to 1.8) |
| 1991 | 1.3 (1.0 to 1.6) | 1997 | 1.4 (1.1 to 1.7) | 2003 | 1.4 (1.1 to 1.8) | 2009 | 1.5 (1.1 to 1.8) | 2015 | 1.5 (1.2 to 1.8) |
| 1992 | 1.3 (1.0 to 1.7) | 1998 | 1.4 (1.1 to 1.7) | 2004 | 1.4 (1.1 to 1.8) | 2010 | 1.5 (1.1 to 1.8) | 2016 | 1.5 (1.2 to 1.8) |
| 1993 | 1.3 (1.1 to 1.7) | 1999 | 1.4 (1.1 to 1.7) | 2005 | 1.4 (1.1 to 1.8) | 2011 | 1.5 (1.1 to 1.8) | 2017 | 1.5 (1.2 to 1.8) |
| 1994 | 1.3 (1.1 to 1.7) | 2000 | 1.4 (1.1 to 1.7) | 2006 | 1.4 (1.1 to 1.8) | 2012 | 1.5 (1.2 to 1.8) | 2018 | 1.5 (1.2 to 1.9) |
| 1995 | 1.3 (1.1 to 1.7) | 2001 | 1.4 (1.1 to 1.8) | 2007 | 1.4 (1.1 to 1.8) | 2013 | 1.5 (1.2 to 1.8) | 2019 | 1.5 (1.2 to 1.9) |
| Brazil | | | | | | | | | |
| 1990 | 1.9 (1.5 to 2.4) | 1996 | 2.0 (1.6 to 2.6) | 2002 | 2.2 (1.7 to 2.8) | 2008 | 2.6 (2.0 to 3.3) | 2014 | 2.9 (2.3 to 3.8) |
| 1991 | 1.9 (1.5 to 2.4) | 1997 | 2.0 (1.6 to 2.6) | 2003 | 2.2 (1.7 to 2.8) | 2009 | 2.7 (2.1 to 3.5) | 2015 | 3.0 (2.3 to 3.8) |
| 1992 | 1.9 (1.5 to 2.5) | 1998 | 2.1 (1.6 to 2.7) | 2004 | 2.2 (1.7 to 2.9) | 2010 | 2.8 (2.2 to 3.6) | 2016 | 3.0 (2.4 to 3.8) |
| 1993 | 1.9 (1.5 to 2.5) | 1999 | 2.1 (1.6 to 2.7) | 2005 | 2.2 (1.8 to 2.9) | 2011 | 2.8 (2.2 to 3.6) | 2017 | 3.0 (2.4 to 3.9) |
| 1994 | 2.0 (1.5 to 2.5) | 2000 | 2.1 (1.6 to 2.7) | 2006 | 2.3 (1.8 to 3.0) | 2012 | 2.9 (2.3 to 3.7) | 2018 | 2.9 (2.3 to 3.7) |
| 1995 | 2.0 (1.6 to 2.6) | 2001 | 2.1 (1.7 to 2.8) | 2007 | 2.4 (1.9 to 3.1) | 2013 | 2.9 (2.3 to 3.7) | 2019 | 2.6 (2.1 to 3.4) |
| Canada | | | | | | | | | |
| 1990 | 3.6 (2.9 to 4.6) | 1996 | 3.8 (3.0 to 4.8) | 2002 | 4.0 (3.1 to 5.1) | 2008 | 4.1 (3.3 to 5.2) | 2014 | 4.2 (3.3 to 5.3) |
| 1991 | 3.6 (2.9 to 4.6) | 1997 | 3.8 (3.0 to 4.8) | 2003 | 4.0 (3.2 to 5.1) | 2009 | 4.1 (3.3 to 5.3) | 2015 | 4.2 (3.3 to 5.4) |
| 1992 | 3.7 (2.9 to 4.6) | 1998 | 3.9 (3.0 to 4.9) | 2004 | 4.0 (3.2 to 5.2) | 2010 | 4.2 (3.3 to 5.3) | 2016 | 4.3 (3.4 to 5.4) |
| 1993 | 3.7 (2.9 to 4.7) | 1999 | 3.9 (3.1 to 4.9) | 2005 | 4.1 (3.2 to 5.2) | 2011 | 4.2 (3.3 to 5.3) | 2017 | 4.3 (3.4 to 5.4) |
| 1994 | 3.7 (3.0 to 4.7) | 2000 | 3.9 (3.1 to 5.0) | 2006 | 4.1 (3.2 to 5.2) | 2012 | 4.2 (3.3 to 5.3) | 2018 | 4.3 (3.4 to 5.4) |
| 1995 | 3.8 (3.0 to 4.7) | 2001 | 4.0 (3.1 to 5.0) | 2007 | 4.1 (3.2 to 5.2) | 2013 | 4.2 (3.3 to 5.3) | 2019 | 4.3 (3.4 to 5.4) |
| Spain | | | | | | | | | |
| 1990 | 3.3 (2.6 to 4.1) | 1996 | 3.5 (2.8 to 4.3) | 2002 | 3.2 (4.0 to 2.7) | 2008 | 2.7 (2.2 to 3.4) | 2014 | 3.7 (2.9 to 4.6) |
| 1991 | 3.3 (2.7 to 4.2) | 1997 | 3.5 (2.8 to 4.3) | 2003 | 3.0 (2.5 to 3.7) | 2009 | 2.7 (2.2 to 3.4) | 2015 | 3.8 (3.0 to 4.7) |
| 1992 | 3.4 (2.7 to 4.2) | 1998 | 3.5 (2.8 to 4.4) | 2004 | 2.7 (2.3 to 3.4) | 2010 | 2.7 (2.2 to 3.4) | 2016 | 3.8 (3.0 to 4.8) |
| 1993 | 3.4 (2.7 to 4.2) | 1999 | 3.5 (2.8 to 4.4) | 2005 | 2.6 (2.2 to 3.3) | 2011 | 2.8 (2.3 to 3.5) | 2017 | 3.8 (3.0 to 4.8) |
| 1994 | 3.4 (2.7 to 4.3) | 2000 | 3.5 (2.8 to 4.4) | 2006 | 2.6 (2.2 to 3.3) | 2012 | 3.1 (2.6 to 3.8) | 2018 | 3.8 (3.1 to 4.8) |
| 1995 | 3.4 (2.7 to 4.3) | 2001 | 3.5 (2.8 to 4.3) | 2007 | 2.7 (2.2 to 3.4) | 2013 | 3.4 (2.8 to 4.3) | 2019 | 3.9 (3.1 to 4.8) |
| Switzerland | | | | | | | | | |
| 1990 | 4.6 (3.7 to 5.7) | 1996 | 4.7 (3.8 to 5.9) | 2002 | 4.8 (3.8 to 6.0) | 2008 | 4.9 (3.9 to 6.1) | 2014 | 4.9 (4.0 to 6.2) |
| 1991 | 4.6 (3.7 to 5.8) | 1997 | 4.7 (3.8 to 5.9) | 2003 | 4.8 (3.9 to 6.0) | 2009 | 4.9 (3.9 to 6.1) | 2015 | 4.9 (4.0 to 6.2) |
| 1992 | 4.6 (3.7 to 5.8) | 1998 | 4.7 (3.8 to 5.9) | 2004 | 4.8 (3.9 to 6.1) | 2010 | 4.9 (3.9 to 6.1) | 2016 | 4.9 (4.0 to 6.2) |
| 1993 | 4.6 (3.7 to 5.8) | 1999 | 4.7 (3.8 to 5.9) | 2005 | 4.8 (3.9 to 6.1) | 2011 | 4.9 (3.9 to 6.2) | 2017 | 4.9 (4.0 to 6.2) |
| 1994 | 4.6 (3.7 to 5.8) | 2000 | 4.7 (3.8 to 6.0) | 2006 | 4.9 (3.9 to 6.1) | 2012 | 4.9 (3.9 to 6.2) | 2018 | 4.9 (4.0 to 6.2) |
| 1995 | 4.7 (3.7 to 5.8) | 2001 | 4.8 (3.8 to 6.0) | 2007 | 4.9 (3.9 to 6.1) | 2013 | 4.9 (4.0 to 6.2) | 2019 | 4.9 (4.0 to 6.2) |
| Abbreviations: *UI*, Uncertainty Interval  ^a^ Prevalence: The proportion of people in a population who are a case of a disease, injury or sequela. All results in GBD refer to point prevalence  Global Burden of Disease Collaborative Network. Global Burden of Disease Study 2019 (GBD 2019) Results. Seattle, United States: Institute for Health Metrics and Evaluation (IHME), 2020. Available from <https://vizhub.healthdata.org/gbd-results/> | | | | | | | | | |

| sTable 7. GBD 2019 modelled prevalence^a^ estimates for knee osteoarthritis in Australia, Brazil, Canada, Spain, and Switzerland from 1990 to 2019. | | | | | | | | | |
| --- | --- | --- | --- | --- | --- | --- | --- | --- | --- |
| Year | **Prevalence estimate % (95% UI)** | **Year** | **Prevalence estimate % (95% UI)** | **Year** | **Prevalence estimate % (95% UI)** | **Year** | **Prevalence estimate % (95% UI)** | **Year** | **Prevalence estimate % (95% UI)** |
| Australia | | | | |  | | | | |
| 1990 | 5.4 (4.6 to 6.2) | 1996 | 6.0 (5.2 to 6.8) | 2002 | 6.6 (5.7 to 7.6) | 2008 | 7.3 (6.3 to 8.4) | 2014 | 7.8 (6.7 to 9.0) |
| 1991 | 5.5 (4.7 to 6.3) | 1997 | 6.1 (5.2 to 7.0) | 2003 | 6.8 (5.9 to 7.8) | 2009 | 7.4 (6.4 to 8.5) | 2015 | 7.9 (6.8 to 9.1) |
| 1992 | 5.6 (4.8 to 6.4) | 1998 | 6.2 (5.3 to 7.1) | 2004 | 6.9 (6.0 to 7.9) | 2010 | 7.5 (6.5 to 8.6) | 2016 | 7.9 (6.8 to 9.2) |
| 1993 | 5.7 (4.9 to 6.5) | 1999 | 6.3 (5.4 to 7.2) | 2005 | 7.0 (6.1 to 8.1) | 2011 | 7.6 (6.5 to 8.7) | 2017 | 8.0 (6.9 to 9.2) |
| 1994 | 5.8 (5.0 to 6.6) | 2000 | 6.4 (5.4 to 7.3) | 2006 | 7.1 (6.2 to 8.2) | 2012 | 7.7 (6.6 to 8.8) | 2018 | 8.1 (7.0 to 9.3) |
| 1995 | 5.9 (5.1 to 6.7) | 2001 | 6.5 (5.6 to 7.5) | 2007 | 7.2 (6.3 to 8.3) | 2013 | 7.7 (6.7 to 8.9) | 2019 | 8.3 (7.2 to 9.5) |
| Brazil | | | | | | | | | |
| 1990 | 2.7 (2.3 to 3.1) | 1996 | 3.0 (2.6 to 3.5) | 2002 | 3.5 (3.0 to 4.0) | 2008 | 4.0 (3.5 to 4.6) | 2014 | 4.7 (4.0 to 5.4) |
| 1991 | 2.7 (2.3 to 3.1) | 1997 | 3.1 (2.6 to 3.5) | 2003 | 3.5 (3.0 to 4.1) | 2009 | 4.1 (3.5 to 4.8) | 2015 | 4.8 (4.1 to 5.5) |
| 1992 | 2.8 (2.4 to 3.2) | 1998 | 3.1 (2.7 to 3.6) | 2004 | 3.6 (3.1 to 4.2) | 2010 | 4.2 (3.6 to 4.9) | 2016 | 4.9 (4.2 to 5.7) |
| 1993 | 2.8 (2.3 to 2.4) | 1999 | 3.2 (2.8 to 3.7) | 2005 | 3.7 (3.2 to 4.3) | 2011 | 4.3 (3.7 to 5.0) | 2017 | 5.0 (4.3 to 5.8) |
| 1994 | 2.9 (2.5 to 3.3) | 2000 | 3.3 (2.8 to 3.8) | 2006 | 3.8 (3.3 to 4.4) | 2012 | 4.5 (3.8 to 5.1) | 2018 | 5.2 (4.4 to 5.9) |
| 1995 | 2.9 (2.5 to 3.4) | 2001 | 3.4 (2.9 to 3.9) | 2007 | 3.9 (3.4 to 4.5) | 2013 | 4.6 (3.9 to 4.3) | 2019 | 5.3 (4.5 to 6.1) |
| Canada | | | | | | | | | |
| 1990 | 3.0 (2.6 to 3.5) | 1996 | 3.4 (2.9 to 3.9) | 2002 | 3.8 (3.3 to 4.4) | 2008 | 4.1 (3.5 to 4.7) | 2014 | 4.5 (3.9 to 5.2) |
| 1991 | 3.1 (2.7 to 3.6) | 1997 | 3.5 (3.0 to 4.0) | 2003 | 3.8 (3.3 to 4.4) | 2009 | 4.2 (3.6 to 4.8) | 2015 | 4.5 (3.9 to 5.2) |
| 1992 | 3.2 (2.7 to 3.7) | 1998 | 3.6 (3.1 to 4.1) | 2004 | 3.9 (3.4 to 4.5) | 2010 | 4.2 (3.6 to 4.9) | 2016 | 4.8 (4.1 to 5.5) |
| 1993 | 3.2 (2.8 to 3.7) | 1999 | 3.6 (3.1 to 4.2) | 2005 | 3.9 (3.4 to 4.5) | 2011 | 4.3 (3.7 to 4.9) | 2017 | 5.0 (4.3 to 5.8) |
| 1994 | 3.3 (2.8 to 3.8) | 2000 | 3.7 (3.2 to 4.3) | 2006 | 4.0 (3.4 to 4.6) | 2012 | 4.3 (3.7 to 5.0) | 2018 | 5.1 (4.3 to 5.9) |
| 1995 | 3.3 (2.9 to 3.9) | 2001 | 3.8 (3.3 to 4.3) | 2007 | 4.0 (3.5 to 4.7) | 2013 | 4.4 (3.8 to 5.1) | 2019 | 5.1 (4.4 to 5.9) |
| Spain | | | | | | | | | |
| 1990 | 5.8 (5.0 to 6.7) | 1996 | 6.5 (5.7 to 7.4) | 2002 | 6.9 (6.1 to 7.8) | 2008 | 7.1 (6.2 to 8.1) | 2014 | 7.8 (6.7 to 8.9) |
| 1991 | 6.0 (5.2 to 6.8) | 1997 | 6.6 (5.8 to 7.5) | 2003 | 6.9 (6.1 to 7.9) | 2009 | 7.2 (6.2 to 8.2) | 2015 | 7.9 (6.8 to 9.0) |
| 1992 | 6.1 (5.3 to 6.9) | 1998 | 6.7 (5.9 to 7.6) | 2004 | 7.0 (6.1 to 7.9) | 2010 | 7.3 (6.3 to 8.3) | 2016 | 8.0 (6.9 to 9.1) |
| 1993 | 6.2 (5.4 to 7.0) | 1999 | 6.8 (6.0 to 7.7) | 2005 | 7.0 (6.1 to 7.9) | 2011 | 7.4 (6.4 to 8.4) | 2017 | 8.1 (7.0 to 9.2) |
| 1994 | 6.3 (5.5 to 7.2) | 2000 | 6.9 (6.0 to 7.7) | 2006 | 7.0 (6.1 to 8.0) | 2012 | 7.5 (6.5 to 8.5) | 2018 | 8.2 (4.1 to 9.3) |
| 1995 | 6.4 (5.6 to 7.3) | 2001 | 6.9 (6.1 to 7.8) | 2007 | 7.1 (6.2 to 8.0) | 2013 | 7.6 (6.6 to 8.7) | 2019 | 8.4 (7.3 to 9.6) |
| Switzerland | | | | | | | | | |
| 1990 | 6.0 (5.2 to 6.9) | 1996 | 6.3 (5.5 to 7.2) | 2002 | 6.7 (5.8 to 7.6) | 2008 | 7.1 (6.1 to 8.1) | 2014 | 7.5 (6.5 to 8.6) |
| 1991 | 6.1 (5.2 to 7.0) | 1997 | 6.4 (5.0 To 7.3) | 2003 | 6.7 (5.8 to 7.7) | 2009 | 7.2 (6.2 to 8.2) | 2015 | 7.6 (6.5 to 8.7) |
| 1992 | 6.1 (5.3 to 7.0) | 1998 | 6.4 (5.5 to 7.4) | 2004 | 6.8 (5.9 to 7.8) | 2010 | 7.2 (6.3 to 8.2) | 2016 | 7.6 (6.6 to 8.7) |
| 1993 | 6.2 (5.4 to 7.1) | 1999 | 6.5 (5.6 to 7.4) | 2005 | 6.9 (6.0 to 7.8) | 2011 | 7.3 (6.3 to 8.3) | 2017 | 7.7 (6.6 to 8.8) |
| 1994 | 6.2 (5.4 to 7.1) | 2000 | 6.5 (5.6 to 7.5) | 2006 | 6.9 (6.0 to 7.9) | 2012 | 7.4 (6.4 to 8.4) | 2018 | 7.8 (6.7 to 8.9) |
| 1995 | 6.3 (5.4 to 7.2) | 2001 | 6.6 (5.7 to 7.6) | 2007 | 7.0 (6.1 to 8.1) | 2013 | 7.4 (6.4 to 8.5) | 2019 | 7.9 (6.8 to 8.9) |
| Abbreviations: *UI*, Uncertainty Interval  ^a^ Prevalence: The proportion of people in a population who are a case of a disease, injury or sequela. All results in GBD refer to point prevalence  Global Burden of Disease Collaborative Network. Global Burden of Disease Study 2019 (GBD 2019) Results. Seattle, United States: Institute for Health Metrics and Evaluation (IHME), 2020. Available from <https://vizhub.healthdata.org/gbd-results/> | | | | | | | | | |

| **sTable 8.** Certainty assessment of the GBD 2019 modelled prevalence estimates of low back pain in Australia, Brazil, Canada, Spain, and Switzerland (1990 to 2019) following GRADE 30 Guidelines [83]. | | | | | | | |
| --- | --- | --- | --- | --- | --- | --- | --- |
| **Certainty assessment** | | | | | | | |
| **Country  (modelling studies^1^)** | **Risk of bias** | **Inconsistency** | **Indirectness** | **Imprecision** | **Other^2^** | **Range of modelled point prevalence estimates from 1990 to 2019 (Range of 95% uncertainty intervals)** | **Overall certainty of modelled evidence** |
| GBD 2019 modelled prevalence estimates of low back pain in Australia, Brazil, Canada, Spain, and Switzerland (1990 to 2019) | | | | | | | |
| Australia  (1 modelling study) | Very serious^a,b^ | Not serious | Not serious | Not serious | None | 11.6 to 12.4 (10.2 to 13.9) | ⨁⨁◯◯  Low^3^ |
| Brazil  (1 modelling study) | Very serious^a,b^ | Not serious | Not serious | Not serious | None | 6.7 to 8.7 (5.8 to 9.9) | ⨁⨁◯◯  Low^3^ |
| Canada  (1 modelling study) | Very serious^a,b^ | Not serious | Not serious | Not serious | None | 10.1 to 12.4 (9.8 to 14.2) | ⨁⨁◯◯  Low^3^ |
| Spain  (1 modelling study) | Very serious^a,b^ | Not serious | Not serious | Not serious | None | 10.1 to 11.1 (9.0 to 12.2) | ⨁⨁◯◯  Low^3^ |
| Switzerland  (1 modelling study) | Very serious^a,b^ | Serious^c^ | Not serious | Not serious | None | 15.0 to 19.2 (13.1 to 20.3) | ⨁◯◯◯  Very Low^4^ |

^1^ Modelling studies were not automatically downgraded and started at “high certainty” by default

^2^ The risk of publication bias in the context of a single model was considered irrelevant and therefore was not included. Domains that could increase the certainty of output from a single model were also considered “nonapplicable”

^3^ Further research is very likely to have an important impact on our confidence in the GBD modelled prevalence estimates for low back pain. Our current confidence in the GBD modelled prevalence estimates is limited

^4^ Any modelled prevalence estimate is very uncertain

**Explanations:**

^a^ The credibility of a model is influenced by its conceptualisation, structure, calibration, validation, and other factors. The GBD 2019 model to estimate the prevalence of LBP involved three main input sources to the nonfatal database: claims data, survey data, and literature data. In addition, it involved the use of a meta-regression—Bayesian, regularized, trimmed (MR-BRT) tool, an adjusted “crosswalk” database, and the feed of location-level covariates (independent variables with a positive or negative relationship to GBD diseases) to the descriptive epidemiological meta-regression (Dismod-MR) tool. Although we had insufficient information to fully assess all dimensions of credibility of the model, we had some concerns about the structure of the GBD LBP model, including the combination of three heterogeneous data types (i.e., claims data, survey data, and literature data) to generate a nonfatal database, the confusing MR-BRT Sex Ratio Analysis to generate modelled age-sex splitting along with the correction analysis for alternative case definitions and methods to generate an adjusted “crosswalk” database, and the application of two arbitrary location-level covariates (derived from ecological measures of occupational ergonomic exposure and increased BMI) to Dismod-MR version 2.1. Hence, we downgraded one level for “credibility of the model”

^b^ The certainty of evidence in each of the model inputs is a critical determinant of the risk of bias in a model, and the model inputs should reflect the entire body of relevant evidence satisfying clear pre-specified criteria. The GBD 2019 model to estimate the prevalence of LBP involved conducting a systematic review from October 2016 to October 2017, which was complemented by additional surveys and opportunistic studies. Because we had insufficient information to assess the input parameters to which the modelled prevalence estimates were the most sensitive, lacked details on the 12 excluded full text articles in the LBP systematic review, and did not find any guidance regarding the criteria considered to include opportunistic additional studies to this systematic review we downgraded one level for “certainty of evidence in each of the model inputs”

^c^ Inconsistency in a single model refers to unexplained variability in modelled outputs. The GBD 2019 modelled prevalence estimates of low back pain in Switzerland decreased from 19.2% (95% UI: 18.2 to 20.3%) in 2015 to 15.0% (13.1 to 17.2%) in 2019, a significant change that could not be explained by any of the primary input studies, which were lacking during this period. In the absence of sensitivity analyses allowing to quantify the degree of inconsistency of model inputs and their influence on the inconsistency of modelled prevalence estimates between years, we found no plausible explanation to the substantial decrease in modelled prevalence estimates in Switzerland between 2015 and 2019 and therefore, downgraded one level for “inconsistency”

| **sTable 9.** Certainty assessment of the GBD 2019 modelled prevalence estimates of neck pain in Australia, Brazil, Canada, Spain, and  Switzerland (1990 to 2019) following GRADE 30 Guidelines [83]. | | | | | | | |
| --- | --- | --- | --- | --- | --- | --- | --- |
| **Certainty assessment** | | | | | | | |
| **Country  (modelling studies^1^)** | **Risk of bias** | **Inconsistency** | **Indirectness** | **Imprecision** | **Other^2^** | **Range of modelled point prevalence estimates from 1990 to 2019 (Range of 95% uncertainty intervals)** | **Overall certainty of modelled evidence** |
| GBD 2019 modelled prevalence estimates of neck pain in Australia, Brazil, Canada, Spain, and Switzerland (1990 to 2019) | | | | | | | |
| Australia  (1 modelling study) | Very serious^a,b^ | Not serious | Serious^c^ | Not serious | None | 1.3 to 1.5 (1.0 to 1.9) | ⨁◯◯◯  Very Low^3^ |
| Brazil  (1 modelling study) | Very serious^a,b^ | Not serious | Not serious | Not serious | None | 1.9 to 3.0 (1.5 to 3.9) | ⨁⨁◯◯  Low^4^ |
| Canada  (1 modelling study) | Very serious^a,b^ | Not serious | Serious^c^ | Not serious | None | 3.6 to 4.3 (2.9 to 5.4) | ⨁◯◯◯  Very Low^3^ |
| Spain  (1 modelling study) | Very serious^a,b^ | Serious^d^ | Not serious | Not serious | None | 2.6 to 3.9 (2.2 to 4.8) | ⨁⨁◯◯  Low^4^ |
| Switzerland  (1 modelling study) | Very serious^a,b^ | Not serious | Serious^c^ | Not serious | None | 4.6 to 4.9 (3.7 to 6.2) | ⨁◯◯◯  Very Low^3^ |

^1^ Modelling studies were not automatically downgraded and started at “high certainty” by default

^2^ The risk of publication bias in the context of a single model was considered irrelevant and therefore was not included. Domains that could increase the certainty of output from a single model were also considered “nonapplicable”

^3^ Any modelled prevalence estimate is very uncertain

^4^ Further research is very likely to have an important impact on our confidence in the GBD modelled prevalence estimates for neck pain. Our current confidence in the GBD modelled prevalence estimates is limited

**Explanations:**

^a^ The credibility of a model is influenced by its conceptualisation, structure, calibration, validation, and other factors. The GBD 2019 model to estimate the prevalence of NP involved three main input sources to the nonfatal database: claims data, survey data, and literature data. In addition, it involved the use of a meta-regression—Bayesian, regularized, trimmed (MR-BRT) tool, and an adjusted “crosswalk” database. Although we had insufficient information to fully assess all dimensions of credibility of the model, we had some certainty concerns about the structure of the GBD NP model, including the combination of three heterogeneous data types (i.e., claims data, survey data, and literature data) to generate a nonfatal database, and the confusing MR-BRT Sex Ratio Analysis to generate modelled age-sex splitting along with the correction analysis for alternative case definitions and methods to generate an adjusted “crosswalk” database. Despite the similarities between the LBP and the NP models, it was unclear why the NP model did not use location-level covariates (independent variables with a positive or negative relationship to GBD diseases) to obtain prevalence estimates. Hence, we downgraded one level for “credibility of the model”

^b^ The certainty of evidence in each of the model inputs is a critical determinant of the risk of bias in a model, and the model inputs should reflect the entire body of relevant evidence satisfying clear pre-specified criteria. The GBD 2019 model to estimate the prevalence of NP involved conducting a systematic review from 2016 to 2017, which was complemented by additional surveys and opportunistic studies. Because we had insufficient information to assess the input parameters to which the modelled prevalence estimates were the most sensitive, lacked details on the 27 excluded full text articles in the NP systematic review, and did not find any guidance regarding the criteria considered to include opportunistic additional studies to this systematic review we downgraded one level for “certainty of evidence in each of the model inputs”

^c^ Assessing indirectness in a single model requires evaluating two separate sources of indirectness: indirectness of model inputs with respect to the model and the indirectness of model outputs with respect to the national MSK modelled prevalence estimation of interest between 1990 and 2019. In countries with no primary data input studies for NP, we found insufficient data to adequately assess the indirectness of model inputs with respect to the model and we assumed that at their best, these modelled prevalence estimates were derived from primary input studies of neighboring countries within the same region. Therefore, we downgraded one level for “indirectness of model inputs with respect to the model”

^d^ Inconsistency in a single model refers to unexplained variability in modelled outputs. The GBD 2019 modelled prevalence estimates of neck pain in Spain changed from 3.5% (95% UI: 2.8 to 4.3%) in 2001 to 2.6% (2.2 to 3.3%) in 2005 and then to 3.7 % (2.9 to 4.6%) in 2014, a significant U-shape change that could not be explained by any of the primary input studies (only one primary input study for Spain for the year 2007). In the absence of sensitivity analyses allowing to quantify the degree of inconsistency of model inputs and their influence on the inconsistency of modelled prevalence estimates between years, we found no plausible explanation to this change in modelled prevalence trends in Spain between 2001 and 2014 and therefore, downgraded one level for “inconsistency”

**sTable 10.** Certainty assessment of the GBD 2019 modelled prevalence estimates of knee osteoarthritis in Australia, Brazil, Canada, Spain, and Switzerland (1990 to 2019) following GRADE 30 Guidelines [83].

| **Certainty assessment** | | | | | | | |
| --- | --- | --- | --- | --- | --- | --- | --- |
| **Country  (modelling studies^1^)** | **Risk of bias** | **Inconsistency** | **Indirectness** | **Imprecision** | **Other^2^** | **Range of modelled point prevalence estimates from 1990 to 2019 (Range of 95% uncertainty intervals)** | **Overall certainty of modelled evidence** |
| GBD 2019 modelled prevalence estimates of knee osteoarthritis in Australia, Brazil, Canada, Spain, and Switzerland (1990 to 2019) | | | | | | | |
| Australia  (1 modelling study) | Very serious^a,b^ | Not serious | Serious^c^ | Not serious | None | 5.4 to 8.3 (4.6 to 9.5) | ⨁◯◯◯  Very Low^3^ |
| Brazil  (1 modelling study) | Very serious^a,b^ | Not serious | Serious^c^ | Not serious | None | 2.7 to 5.4 (2.3 to 6.1) | ⨁◯◯◯  Very Low^3^ |
| Canada  (1 modelling study) | Very serious^a,b^ | Not serious | Not serious | Not serious | None | 3.0 to 5.1 (2.6 to 5.9) | ⨁⨁◯◯  Low^4^ |
| Spain  (1 modelling study) | Very serious^a,b^ | Not serious | Not serious | Not serious | None | 5.8 to 8.4 (5.0 to 9.6) | ⨁⨁◯◯  Low^4^ |
| Switzerland  (1 modelling study) | Very serious^a,b^ | Not serious | Serious^c^ | Not serious | None | 6.0 to 7.9 (5.2 to 8.9) | ⨁◯◯◯  Very Low^3^ |

^1^ Modelling studies were not automatically downgraded and started at “high certainty” by default

^2^ The risk of publication bias in the context of a single model was considered irrelevant and therefore was not included. Domains that could increase the certainty of output from a single model were also considered “nonapplicable”

^3^ Any modelled prevalence estimate is very uncertain

^4^ Further research is very likely to have an important impact on our confidence in the GBD modelled prevalence estimates for knee osteoarthritis. Our current confidence in the GBD modelled prevalence estimates is limited

**Explanations:**

^a^ The credibility of a model is influenced by its conceptualisation, structure, calibration, validation, and other factors. The GBD 2019 model to estimate the prevalence of knee OA involved three main input sources to the nonfatal database: claims data, survey data, and literature data. In addition, it involved the use of a meta-regression—Bayesian, regularized, trimmed (MR-BRT) tool, an adjusted “crosswalk” database, and the feed of predictive covariates (independent variables with a positive or negative relationship to GBD diseases) to the descriptive epidemiological meta-regression (Dismod-MR) tool. Although we had insufficient information to fully assess all dimensions of credibility of the model, we had some concerns about the structure of the GBD knee OA model, including the combination of three heterogeneous data types (i.e., claims data, survey data, and literature data) to generate a nonfatal database, the confusing MR-BRT Sex Ratio Analysis to generate modelled age-sex splitting along with the correction analysis for alternative case definitions and methods to generate an adjusted “crosswalk” database, and the application of an arbitrary location-level covariate (derived from ecological measures of mean body mass index) to Dismod-MR version 2.1. Hence, we downgraded one level for “credibility of the model”

^b^ The certainty of evidence in each of the model inputs is a critical determinant of the risk of bias in a model, and the model inputs should reflect the entire body of relevant evidence satisfying clear pre-specified criteria. The GBD 2019 model to estimate the prevalence of knee OA involved conducting a systematic review from 1980 to 2019, which was complemented by additional studies. Because we had insufficient information to assess the input parameters to which the modelled prevalence estimates were the most sensitive, lacked details on the 237 excluded full text articles in the knee OA systematic review, and did not find any guidance regarding the criteria considered to include additional studies to this systematic review we downgraded one level for “certainty of evidence in each of the model inputs”

^c^ Assessing indirectness in a single model requires evaluating two separate sources of indirectness: indirectness of model inputs with respect to the model and the indirectness of model outputs with respect to the national MSK modelled prevalence estimation of interest between 1990 and 2019. In countries with no primary data input studies for knee OA, we found insufficient data to adequately assess the indirectness of model inputs with respect to the model and we assumed that at their best, these modelled prevalence estimates were derived from primary input studies of neighboring countries within the same region. Therefore, we downgraded one level for “indirectness of model inputs with respect to the model”

**References**

1. Hoy D, Bain C, Williams G, March L, Brooks P, Blyth F, et al. A systematic review of the global prevalence of low back pain. Arthritis Rheum. 2012 Jun;64(6):2028–37.

2. Hoy D, Brooks P, Woolf A, Blyth F, March L, Bain C, et al. Assessing risk of bias in prevalence studies: modification of an existing tool and evidence of interrater agreement. J Clin Epidemiol. 2012 Sep 1;65(9):934–9.

3. Ramelow D, Griebler R, Hofmann F, Unterweger K, Mager U, Felder-Puig R, et al. Gesundheit und Gesundheitsverhalten von österreichischen Schülern und Schülerinnen Ergebnisse des WHO-HBSC-Survey 2010. Bundesministerium für Gesundheit, Sekt. III [Internet]. 2011. Available from: http://www.hbsc.org/membership/countries/austria.html

4. Balagué F, Nordin M, Skovron ML, Dutoit G, Yee A, Waldburger M. Non-specific low-back pain among schoolchildren: a field survey with analysis of some associated factors. J Spinal Disord. 1994 Oct;7(5):374–9.

5. McLennan W. National Health Survey. User’s guide. Cat. no. 4363.0. Australian Bureau of Statistics, Canberra, 1995.

6. National Health Survey. Summary of results. Cat. no. 4364.0. Australian Bureau of Statistics, Canberra, 1995.

7. National Health Survey. User’s guide. Cat. no. 4363.0.55.001. Australian Bureau of Statistics, Canberra, 2001.

8. National Health Survey. Summary of results. Cat. no. 4364.0. Australian Bureau of Statistics, Canberra, 2001.

9. Trewin D. Survey of Disability, Ageing and Carers. Users’ guide. Cat. no. 4431.0.55.001. Australian Bureau of Statistics, Canberra, 2003.

10. Survey of Disability, Ageing and Carers. Summary of findings. Cat. no. 4430.0. Australian Bureau of Statistics, Canberra, 2003.

11. World Health Organization. World Health Survey 2003. Report of Australia.

12. Walker BF, Muller R, Grant WD. Low back pain in Australian adults. Prevalence and associated disability. J Manipulative Physiol Ther. 2004 May 1;27(4):238–44.

13. National Health Survey. User’s guide. Cat. no. 4363.0.55.001. Australian Bureau of Statistics, Canberra, 2004-2005.

14. National Health Survey. Summary of results. Cat. no. 4364.0. Australian Bureau of Statistics, Canberra, 2004-2005.

15. Grimmer K, Nyland L, Milanese S. Longitudinal investigation of low back pain in Australian adolescents: a five-year study. Physiother Res Int. 2006;11(3):161–72.

16. National Health Survey. User’s guide. Cat. no. 4363.0.55.001. Australian Bureau of Statistics, Canberra, 2007-2008.

17. National Health Survey. Summary of results. Cat. no. 4364.0. Australian Bureau of Statistics, Canberra, 2007-2008 (Reissue).

18. Pink B. Survey of Disability, Ageing and Carers. Users’ guide. Cat. no. 4431.0.55.001. Australian Bureau of Statistics, Canberra, 2009.

19. Survey of Disability, Ageing and Carers. Summary of findings. Cat. no. 4431.0.55.001. Australian Bureau of Statistics, Canberra, 2009.

20. Broom AF, Kirby ER, Sibbritt DW, Adams J, Refshauge KM. Back pain amongst mid-age Australian women: a longitudinal analysis of provider use and self-prescribed treatments. Complement Ther Med. 2012 Oct;20(5):275–82.

21. O’Sullivan PB, Beales DJ, Smith AJ, Straker LM. Low back pain in 17 year olds has substantial impact and represents an important public health disorder: a cross-sectional study. BMC Public Health. 2012 Feb 5;12(1):100.

22. World Health Organization. World Health Survey 2003. Report of Brazil.

23. Silva MC da, Fassa AG, Valle NCJ. Chronic low back pain in a Southern Brazilian adult population: prevalence and associated factors. Cad Saude Publica. 2004 Apr;20(2):377–85.

24. Mendoza-Sassi R, Béria JU, Fiori N, Bortolotto A. Prevalence of signs and symptoms, associated sociodemographic factors and resulting actions in an urban center in southern Brazil. Pan Am J Public Health. 2006 Jul;20(1):22–8.

25. Blay SL, Andreoli SB, Dewey ME, Gastal FL. Co-occurrence of chronic physical pain and psychiatric morbidity in a community sample of older people. Int J Geriatr Psychiatry. 2007;22(9):902–8.

26. De Vitta A, Martinez MG, Piza NT, Simeão SF de AP, Ferreira NP. Prevalence of lower back pain and associated factors in students. Cad Saude Publica. 2011 Aug;27(8):1520–8.

27. Ferreira GD, Silva MC, Rombaldi AJ, Wrege ED, Siqueira FV, Hallal PC. Prevalence and associated factors of back pain in adults from southern Brazil: a population-based study. Rev Bras Fisioter. 2011 Feb;15(1):31–6.

28. Onofrio AC, da Silva MC, Domingues MR, Rombaldi AJ. Acute low back pain in high school adolescents in Southern Brazil: prevalence and associated factors. Eur Spine J. 2012 Jul 1;21(7):1234–40.

29. Meziat Filho N, Coutinho ES, Azevedo e Silva G. Association between home posture habits and low back pain in high school adolescents. Eur Spine J. 2015 Mar 1;24(3):425–33.

30. Depintor JDP, Bracher ESB, Cabral DMC, Eluf-Neto J. Prevalence of chronic spinal pain and identification of associated factors in a sample of the population of São Paulo, Brazil: cross-sectional study. Sao Paulo Med J. 2016 Oct;134(5):375–84.

31. Noll M, Candotti CT, Rosa BN da, Loss JF. Back pain prevalence and associated factors in children and adolescents: an epidemiological population study. Rev Saude Publica. 2016 Jun 10;50:S0034-89102016000100220.

32. Liira JP, Shannon HS, Chambers LW, Haines TA. Long-term back problems and physical work exposures in the 1990 Ontario Health Survey. Am J Public Health. 1996 Mar;86(3):382–7.

33. HBSC Canada 1997/1998. Survey Data. Available from: https://www.canada.ca/content/dam/phac-aspc/migration/phac-aspc/hp-ps/dca-dea/prog-ini/school-scolaire/behaviour-comportements/publications/pdf/hbsc97983gr_ef.pdf.

34. Currie C, Hurrelmann K, Settertobulte W, Smith R, Todd J. Health and health behaviour among young people in school-aged children: international report from the HBSC 1997/1998 survey. WHO policy series: health policy for children and adolescents, Issue 1. Copenhagen, Denmark: WHO Regional Office for Europe. World Health Organization Regional Office for Europe; 2000.

35. Cassidy JD, Carroll LJ, Côté P. The Saskatchewan health and back pain survey. The prevalence of low back pain and related disability in Saskatchewan adults. Spine. 1998 Sep 1;23(17):1860–6.

36. HBSC Canada 2001/2002. Statistical tables. Available from: https://www.canada.ca/content/dam/phac-aspc/migration/phac-aspc/hp-ps/dca-dea/prog-ini/school-scolaire/behaviour-comportements/publications/pdf/hbsc_2001_2001.pdf.

37. Currie C, Roberts C, Morgan A, Smith R, Settertobulte W, Samdal O, et al. Young people’s health in context: international report from the HBSC 2001/2002 survey. WHO policy series: health policy for children and adolescents, Issue 4. Copenhagen, Denmark: WHO Regional Office for Europe; 2004.

38. Currie SR, Wang J. Chronic back pain and major depression in the general Canadian population. PAIN. 2004 Jan;107(1):54–60.

39. Public Health Agency of Canada. Healthy settings for young people in Canada. 2008. Available from: https://www.canada.ca/en/public-health/services/health-promotion/childhood-adolescence/publications/healthy-settings-young-people-canada.html.

40. Currie C, Nic Gabhainn S, Godeau E. Inequalities in young people’s health: International report from the HBSC 2005/2006 survey. WHO policy series: health policy for children and adolescents, Issue 5. Copenhagen, Denmark: WHO Regional Office for Europe; 2008.

41. Public Health Agency of Canada. The health of Canada’s young people: a mental health focus. 2012. Available from: https://www.canada.ca/en/public-health/services/health-promotion/childhood-adolescence/publications/health-canada-young-people-mental-health-focus.html.

42. Currie C, Zanotti C, Morgan A. Social determinants of health and well-being among young people: international report from the HBSC 2009/2010 survey. WHO policy series: health policy for children and adolescents, Issue 6. Copenhagen, Denmark: WHO Regional Office for Europe; 2012.

43. Ballina Garcia FJ, Hernandez Mejía R, Martín Lascuevas P, Fernandez Santana J, Cueto Espinar A. Epidemiology of musculoskeletal complaints and use of health services in Asturias, Spain. Scand J Rheumatol. 1994;23(3):137–41.

44. Carmona L, Ballina J, Gabriel R, Laffon A. The burden of musculoskeletal diseases in the general population of Spain: results from a national survey. Ann Rheum Dis. 2001 Nov;60(11):1040–5.

45. Sociedad Española de Reumatología (2006). Estudio EPISER. Prevalencia e impacto de las enfermedades reumáticas en la población adulta española. Madrid.

46. Català E, Reig E, Artés M, Aliaga L, López JS, Segú JL. Prevalence of pain in the Spanish population telephone survey in 5000 homes. Eur J Pain. 2002;6(2):133–40.

47. Moreno Rodríguez MC, Muñoz V, Pérez P, Sánchez Queija I. Los adolescentes españoles y su salud. Resumen del estudio “Health Behaviour in School Aged Children (HBSC-2002)”. (Colección “salud pública: promoción de la salud y epidemiología”). Madrid: Ministerio de Sanidad y Consumo. 2005.

48. World Health Organization. World Health Survey 2003. Report of Spain.

49. Pinto-Meza A, Serrano-Blanco A, Codony M, Reneses B, von Korff M, Maria Haro J, et al. Prevalencia y comorbilidad física y mental del dolor dorsal y cervical crónicos en España: resultados del estudio ESEMeD. Med Clínica. 2006 Sep 1;127(9):325–30.

50. Demyttenaere K, Bruffaerts R, Lee S, Posada-Villa J, Kovess V, Angermeyer MC, et al. Mental disorders among persons with chronic back or neck pain: Results from the world mental health surveys. PAIN. 2007 Jun;129(3):332–42.

51. Alonso J, Angermeyer MC, Bernert S, Bruffaerts R, Brugha TS, Bryson H, et al. Sampling and methods of the European Study of the Epidemiology of Mental Disorders (ESEMeD) project. Acta Psychiatr Scand Suppl. 2004;(420):8–20.

52. Miró J, Paredes S, Rull M, Queral R, Miralles R, Nieto R, et al. Pain in older adults: a prevalence study in the Mediterranean region of Catalonia. Eur J Pain Lond Engl. 2007 Jan;11(1):83–92.

53. Pellisé F, Balagué F, Rajmil L, Cedraschi C, Aguirre M, Fontecha CG, et al. Prevalence of low back pain and its effect on health-related quality of life in adolescents. Arch Pediatr Adolesc Med. 2009 Jan;163(1):65–71.

54. Moreno C, Ramos P, Rivera F, Jiménez-Iglesias A, García Moya I, Sánchez-Queija I, et al. Las conductas relacionadas con la salud y el desarrollo de los adolescentes españoles. Resultados del estudio HBSC-2010 con chicos y chicas españoles de 11 a 18 años. 2012;920.

55. Fernández-de-las-Peñas C, Hernández-Barrera V, Alonso-Blanco C, Palacios-Ceña D, Carrasco-Garrido P, Jiménez-Sánchez S, et al. Prevalence of neck and low back pain in community-dwelling adults in Spain: a population-based national study. Spine. 2011 Feb 1;36(3):E213-219.

56. Instituto Nacional de Estadística. Encuesta Nacional de Salud 2006. Metodología detallada. Madrid: Instituto Nacional de Estadística y Ministerio de Sanidad y Política Social; 2007.

57. Instituto Nacional de Estadística. Encuesta Nacional de Salud 2009. Evaluación de la falta de respuesta en la Encuesta Nacional de Salud 2009; 2010.

58. Balagué F, Ferrer M, Rajmil L, Pont Acuña A, Pellisé F, Cedraschi C. Assessing the association between low back pain, quality of life, and life events as reported by schoolchildren in a population-based study. Eur J Pediatr. 2012 Mar;171(3):507–14.

59. Papacostas A. Eurobarometer 66.2: nuclear energy and safety, and public health issues. In: ICPSR21460-v2 (ed). GESIS/Ann Arbor, MI: Inter-university Consortium for Political and Social Research Cologne, Germany.

60. Papacostas A. Eurobarometer 66.2: nuclear energy and safety, and public health issues. Codebook. In: ICPSR21460-v2 (ed). GESIS/Ann Arbor, MI: Inter-university Consortium for Political and Social Research Cologne, Germany.

61. Jiménez-Sánchez S, Fernández-de-Las-Peñas C, Carrasco-Garrido P, Hernández-Barrera V, Alonso-Blanco C, Palacios-Ceña D, et al. Prevalence of chronic head, neck and low back pain and associated factors in women residing in the Autonomous Region of Madrid (Spain). Gac Sanit. 2012 Dec;26(6):534–40.

62. Rodríguez Laso Á, Ramasco Gutiérrez M, Cruz Maceín JL, Rodríguez Rieiro C, Garabato González S, Aerny Perreten N. Informe sobre desigualdades en salud a partir de la Encuesta Regional de Salud de Madrid 2007. 2010.

63. Rodríguez-Oviedo P, Ruano-Ravina A, Pérez-Ríos M, García FB, Gómez-Fernández D, Fernández-Alonso A, et al. School children’s backpacks, back pain and back pathologies. Arch Dis Child. 2012 Aug;97(8):730–2.

64. Vargas-Prada S, Serra C, Martínez JM, Ntani G, Delclos GL, Palmer KT, et al. Psychological and culturally-influenced risk factors for the incidence and persistence of low back pain and associated disability in Spanish workers: findings from the CUPID study. Occup Environ Med. 2013 Jan;70(1):57–62.

65. Mesas AE, González AD, Mesas CE, de Andrade SM, Magro IS, del Llano J. The association of chronic neck pain, low back pain, and migraine with absenteeism due to health problems in Spanish workers. Spine. 2014 Jul 1;39(15):1243–53.

66. Koyanagi A, Stickley A, Garin N, Miret M, Ayuso-Mateos JL, Leonardi M, et al. The association between obesity and back pain in nine countries: a cross-sectional study. BMC Public Health. 2015 Feb 11;15:123.

67. Lara E, Miret M, Olaya B, Caballero FF, Morillo D, Moneta MV, et al. Cohort Profile: The Spanish Longitudinal Study on Ageing and Health (Edad Con Salud). Int J Epidemiol. 2022 Jun 8;dyac118.

68. Tillmann R, Voorpostel M, Antal E, Dasoki N, Klaas H, Kuhn U, et al. The Swiss Household Panel (SHP). Jahrb Für Natl Stat. 2021 Nov 3;242(3):403–20.

69. Voorpostel M, Tillmann R, Lebert F, Kuhn U, Lipps O, Ryser VA, et al. Swiss Household Panel User Guide (1999 - 2016). Lausanne, FORS.

70. Santos-Eggimann B, Wietlisbach V, Rickenbach M, Paccaud F, Gutzwiller F. One-year prevalence of low back pain in two Swiss regions: estimates from the population participating in the 1992-1993 MONICA project. Spine. 2000 Oct 1;25(19):2473–9.

71. Ambord S, Eichenberger Y, Delgrande Jordan M. Gesundheit und Wohlbefinden der 11- bis 15-jährigen Jugendlichen in der Schweiz im Jahr 2018 und zeitliche Entwicklung - Resultate der Studie “Health Behaviour in School-aged Children” (HBSC) (Forschungsbericht Nr. 113). Lausanne: Sucht Schweiz. Lausanne: Addiction Suisse.

72. Delgrande Jordan M, Annaheim B. Habitudes alimentaires, activité physique et statut pondéral chez les élèves de 11 à 15 ans en Suisse. Situation en 2006 et évolution récente - Résultats de l’Enquête internationale Health Behaviour in School-aged Children (HBSC). Lausanne: Institut suisse de prévention de l’alcoolisme et autres toxicomanies (ISPA). 2009.

73. Delgrande Jordan M. Multipler Substanzkonsum Systematische Zusammenstellung von Prävalenzen bei 11- bis 15-jährigen Jugendlichen in der Schweiz. Resultate der internationalen Studie «Health Behaviour in School-aged Children» (HBSC) 2010. Lausanne, Mai 2013; Report No.: Forschungsbericht Nr. 66.

74. Erne C, Elfering A. Low back pain at school: unique risk deriving from unsatisfactory grade in maths and school-type recommendation. Eur Spine J. 2011 Dec;20(12):2126–33.

75. Kolb E, Canjuga M, Bauer GF, Läubli T. Course of back pain across 5 years: A retrospective cohort study in the general population of Switzerland. Spine. 2011 Feb 15;36(4):E268.

76. Genebra CVDS, Maciel NM, Bento TPF, Simeão SFAP, Vitta AD. Prevalence and factors associated with neck pain: a population-based study. Braz J Phys Ther. 2017;21(4):274–80.

77. de Barros ENC, Alexandre NMC. Cross-cultural adaptation of the Nordic musculoskeletal questionnaire. Int Nurs Rev. 2003 Jun;50(2):101–8.

78. Plotnikoff R, Karunamuni N, Lytvyak E, Penfold C, Schopflocher D, Imayama I, et al. Osteoarthritis prevalence and modifiable factors: a population study. BMC Public Health. 2015 Nov 30;15:1195.

79. Raine KD, Plotnikoff R, Schopflocher D, Lytvyak E, Nykiforuk CIJ, Storey K, et al. Healthy Alberta Communities: Impact of a three-year community-based obesity and chronic disease prevention intervention. Prev Med. 2013 Dec 1;57(6):955–62.

80. Altman R, Asch E, Bloch D, Bole G, Borenstein D, Brandt K, et al. Development of criteria for the classification and reporting of osteoarthritis: Classification of osteoarthritis of the knee. Arthritis Rheum. 1986;29(8):1039–49.

81. Fernandez-Lopez JC, Laffon A, Blanco FJ, Carmona L, EPISER Study Group. Prevalence, risk factors, and impact of knee pain suggesting osteoarthritis in Spain. Clin Exp Rheumatol. 2008 Apr;26(2):324–32.

82. McGuinness LA, Higgins JPT. Risk-of-bias VISualization (robvis): An R package and Shiny web app for visualizing risk-of-bias assessments. Res Synth Methods. 2021;12(1):55–61.

83. Brozek JL, Canelo-Aybar C, Akl EA, Bowen JM, Bucher J, Chiu WA, et al. GRADE Guidelines 30: the GRADE approach to assessing the certainty of modeled evidence—An overview in the context of health decision-making. J Clin Epidemiol. 2021 Jan 1;129:138–50.
